# A comprehensive review on bacterial endophytic secondary metabolites: a road map from crude extract to lead molecule production

**DOI:** 10.3389/fphar.2026.1837576

**Published:** 2026-07-16

**Authors:** Reshma Biju, W. Jabez Osborne

**Affiliations:** School of Bio Sciences and Technology, Vellore Institute of Technology, Vellore, Tamil Nadu, India

**Keywords:** downstream process, high-throughput technologies, pathogenicity islands, single metabolite, toxicity assessment, upstream process

## Abstract

Endophytes are microorganisms that are concealed within the intracellular spaces of the plants in diverse ecological niches across varied habitats, are a prolific source of bioactive metabolites capable of inhibiting human pathogens responsible for mild to severe infections. The rising threat of antimicrobial resistance largely due to the indiscriminate use of antibiotics, has prompted the World Health Organization to advocate for rigorous stewardship of existing drugs while simultaneously urging the development of novel therapeutic alternatives. Several studies have reported emphasizing the application of multiple metabolites from endophytes however, exploring the mechanisms involved in the inhibition by a single effective metabolite which could help in unwinding the complexity during lead molecule synthesis. This review aims to present a comprehensive and strategic roadmap integrating upstream processes encompassing isolation, screening, and fermentation, with downstream methodologies for metabolite extraction, purification and structural elucidation, along with toxicity assessments for effective pharmaceutical applications. Furthermore, the discussion also considers the integration of predictive AI-ML models in accelerating the drug developmental pipeline for circumventing the traditional discovery bottlenecks highlighting the relevance to industrial translation and underlines the potential of endophyte-derived metabolites to target pathogenicity islands, offering a paradigm shift from bacterial eradication to anti-virulence strategies along with a focus on errors and limitations of endophytic research.

## Graphical Abstract

**Figure:**
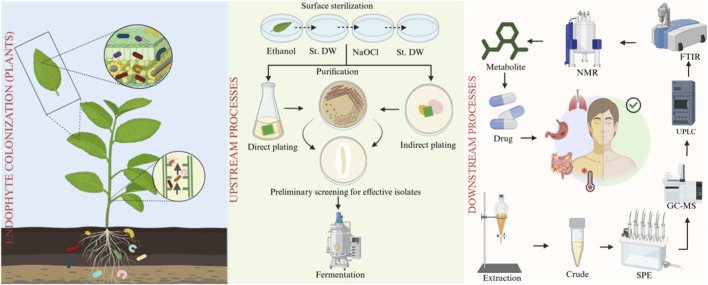
Created in BioRender. Osborne, J. (2026) https://BioRender.com/r3zwi5r.

## 1 Introduction

Reducing infection-related mortality remains a global health priority (Ikuta et al., 2022). Despite their indispensability, antibiotic access remains limited in many low- and middle-income countries (LMICs), contributing to 7.7 million annual deaths from bacterial infections. Simultaneously, the indiscriminate and unprescribed use of antibiotics has accelerated the emergence of antimicrobial resistance (AMR), thereby compromising the efficacy of existing therapeutic agents (Elshobary et al., 2025; Balasegaram and Muñoz et al., 2024). In this context, implementation of universal antibiotic stewardship programs has been projected to reduce infection-related mortality by nearly 10% by 2030 (Balasegaram and Muñoz Tellez, 2024). Considering the escalating threat posed by resistant pathogens, the World Health Organization (WHO) has identified AMR as one of the top ten global health threats (Elshobary et al., 2025). Among these, multi-drug resistant (MDR) ESKAPE (*Enterococcus faecium, Staphylococcus aureus, Klebsiella pneumoniae, Acinetobacter baumannii, Pseudomanas aeruginosa, and Enterobacter species*) pathogens recognized under Bacterial Priority Pathogen List (BPPL) since 2017 have contributed significantly to global health crisis, accounting for approximately 700,000 deaths annually (Sakalauskienė et al., 2025; Pasrija et al., 2022), emphasizing the need for novel therapeutic strategies (Elshobary et al., 2025).

Literature reports indicate that the emergence of resistant bacteria is increasing at a rate faster than the development of new antibiotics. Between 2010 and 2014, only four novel antibiotics were approved, while from 2014 to 2021, only 18 antibiotics received approval, among which only two possessed novel mechanisms of action. Furthermore, most recently approved antibiotics are merely structural modification of existing drug classes rather than entirely novel compounds (Liu G. Y. et al., 2024). These data highlight the inability of the current antibiotic discovery pipeline to meet the rapidly growing demand for new therapeutics. Acoltremon, Nafithromycin, Xanomeline, Cantharidin are all some of the natural product derived drugs mainly launched in market between 2023–2025 (Butler et al., 2026). Drug abuse on the other hand is making the currently available antibiotics ineffective (Wang, 2026). Also, studies have reported that failure of drugs at preclinical and clinical trial attributes to 30% (Zhang J. et al., 2025), thus revealing the inefficiency of existing drug developmental pipeline in producing stable drugs with high bioactive potential. Early safety profile assessment and toxicity evaluations coupled with pharmacodynamic, and pharmacokinetic properties might reduce this loophole in the current drug developmental pipeline.

Medicinal plants have been recognized for ages as important reservoirs of natural bioactive molecules with the potential for novel drug development. However, exploiting endophytic bacteria capable of producing bioactive metabolites is considered a more sustainable approach than direct extraction from plants through continuous harvesting (Lata and Gond, 2025). Endophytes are microorganisms initially present in external environment of the plant that become recruited into plant tissues, where they establish asymptomatic intra and intercellular colonization and contribute to plant defense mechanisms. Certain well-adapted microbes are transmitted to subsequent generations thereby establishing a stable endophytic microbial community within the host plant (Zhao et al., 2024).

Traditionally endophytes were defined as microorganisms that reside within the intracellular spaces of the plant without causing harm to the host plant, isolated from internal tissues upon surface-sterilization of plant tissues. However, with the advancement of metagenomic and molecular approaches targeting unculturable microbial communities, this definition is now insufficient as highly sensitive molecular techniques can also detect transient soil epiphytes surviving inadequate surface sterilization or latent phytopathogens existing in dormant states (Hardoim et al., 2015). Therefore, endophytes can be redefined as microbes that reside within the plant tissues without causing any damage to host, establish a stable colonization and supports a stable intra and inter cellular localization rather than mere recovery followed by surface sterilization.

Endophyte derived metabolites, including terpenes, siderophores, non-ribosomal peptides, flavonoids, phenolics, and alkaloids, exhibit strong activity against multi-drug resistant (MDR) pathogens (Akter et al., 2022; Watts et al., 2023). Moreover, endophytes can also function as antibiotic adjuvants, enhancing the control of MDR pathogens (Moussa, 2024). Owing to the remarkable efficiency of endophytic bacterial metabolites, numerous studies have been reported focusing on the identification and purification of bioactive metabolites for drug development against human pathogens. Despite the strong efficacy of novel metabolites derived from bacterial endophytes, the existing metabolite elucidation and drug development pipelines are associated with several limitations. The rediscovery of known metabolites results in significant loss of time and resources, particularly in the absence of early stage dereplication strategies. Furthermore, difficulties in compound identification and failure during later preclinical studies considerably delay the drug discovery process.

Although a standard percentage for metabolite rediscovery is not currently available, the pronounced escalation in dereplication focused research clearly signifies the severity of redundancy within conventional natural product discovery workflows. Gaudêncio et al. (2023) revealed that nearly 1,240 publications in the field of dereplication including 908 articles published after April 2014 have collectively received 40,520 citations. This strongly emphasizes metabolite rediscovery rates as a translational hurdle. Attrition of lead compounds is frequently reported in drug discovery, with approximately 30%–70% occurring at the preclinical stage (Kramer et al., 2007; Zhang J. et al., 2025). This is largely attributed to inadequate early-stage toxicity assessment, resulting in loss of potentially valuable crude extracts and bioactive metabolites, which are available only in limited quantities. Consequently, researchers are frequently compelled to repeat fermentation processes to obtain sufficient amounts of the desired compounds which further contributes to delayed development. Although preclinical attrition cannot be eliminated completely it can be minimized through early-stage toxicity assessment and integrating AI and ML models to create predictions. In addition to these limitations, methodological inconsistencies persist within the existing pipeline, thereby necessitating the development of enhanced metabolomics and AI-ML based approaches for efficient natural product drug discovery.

Recent studies (2023–2025) show greater advancement in endophytic fungal research compared to bacterial counterparts. For instance, *Sargassum sp*. derived fungal endophytes exhibited potent antibacterial activity, with GC-MS revealing diverse bioactive metabolites (Noor et al., 2025). Likewise, *Tinospora cordifolia* derived fungal endophytes were extensively characterized using HRESI-MS, GC-MS and FT-IR for confirming the presence of diverse pharmacologically active compounds (Uzma et al., 2024). In contrast, bacterial endophyte studies, such as those from *Calotropis gigantea* and *Acacia nilotica* (Chauhan et al., 2025) or salt-tolerant barley (Hussein et al., 2024), largely remained at crude extract evaluation without progressing to purification or structural elucidation. Only a few, like the isolation of plicacetin from *Streptomyces sp.* (Devi et al., 2023) have reached full downstream characterization, underscoring the limited development in bacterial endophyte research relative to fungi. The extensive investigation of fungal metabolites may be attributed to early identification of clinically important compounds like penicillin and cephalosporins (Keller et al., 2005), which naturally directed early natural product research towards fungal endophytes. However, recent advances in omics technologies have increasingly revealed the bioactive potential of bacterial endophytes for bioactive production thereby expanding research focus towards bacterial endophytes.

Furthermore, combinations of multiple metabolites derived from same or different endophytes have been reported to exhibit enhanced synergistic activity as compared to individual metabolites (Liao et al., 2024; Elfita et al., 2024). Although such interactions enhance efficacy, purification of individual metabolites remains inevitable to avoid false-positive or misinterpreted results arising from mixed-compound interference (Queiroz et al., 2024). Moreover, when synergistic activity is exhibited by multiple metabolites produced from the same endophyte, the individual contribution and relative efficacy of each metabolite may remain unclear without purification and separate evaluation. Therefore, purification of single effective metabolites is important for accurate assessment of bioactivity and further efficacy optimization.

Pharmaceutical production begins with upstream bioprocessing, encompassing isolation of microorganisms to biomass harvest, while downstream processing involves the recovery and purification of target biomolecules after fermentation (Yasu, 2024; Shanu et al., 2024; Agarwal et al., 2020). Isolation represents a critical upstream step, requiring the removal of epiphytic contaminants through surface sterilization with agents such as ethanol or sodium hypochlorite, optimized according to host tissue type (Srivastava et al., 2024). Discovery of novel antimicrobial metabolites employs diverse assays, disc diffusion, agar and broth dilution, well diffusion, cross-streak, and gradient-based methods, each with specific advantages and limitations that demand methodological precision (Hossain et al., 2024). Once bioactivity is established, fermentation is essential for yield enhancement and economic viability. Shake-flask studies typically are used for bioreactor optimization, enabling effective scale-up for industrial production (Mendes et al., 2024).

Following fermentation, downstream processing focuses on metabolite recovery and purification from complex matrices. Among extraction methods, liquid-liquid extraction (LLE) is favored for its simplicity, scalability, and polarity-based selectivity (Dey et al., 2023; Manam et al., 2025a), while solid-phase extraction (SPE) offers further refinement (Badawy et al., 2022). Purified fractions are analyzed using GC-MS, LC-HRMS, UPLC/HPLC for separation and quantification, and FT-IR and NMR for functional and structural elucidation (Manam et al., 2025a; Sriwastava and Srivastava, 2021; Veilumuthu et al., 2024). Toxicity screening through *in vitro* assays such as MTT and XTT, along with *in vivo* seed germination assays, ensures biosafety prior to advanced evaluations. Integration of pharmacokinetic and pharmacodynamic (PK/PD) analyses bridges *in vitro* potency and *in vivo* efficacy, optimizing dosage and minimizing adverse effects (Alikhani et al., 2025).

This review provides a comprehensive roadmap for endophyte-derived natural product discovery, spanning isolation, screening, fermentation, and purification focusing on single metabolite identification to aid the rational drug discovery pipeline. It further emphasizes analytical workflows, cytotoxicity evaluation, and PK/PD profiling, while highlighting bacterial virulence mechanisms and pathogenicity islands as emerging targets for next-generation anti-infective development. The review also sheds light on limitations of endophytic research till date and the future prospects of endophytes in pharmaceuticals while emphasizing the importance of integrating AI-ML models in active pharmaceutical ingredient (API) development to significantly accelerate the drug developmental pipeline.

## 2 Endophytic sources of active pharmaceutical ingredients (API) for the inhibition of human pathogens

Building upon the growing interest in fungal endophytes, bacterial endophytes are now gaining attention as underexplored reservoirs of lead molecules with potential as APIs. Their ease of isolation, rapid growth, and adaptability under laboratory conditions make them suitable for large-scale metabolite production and characterization (Semenzato et al., 2024). These endophytic microorganisms synthesize a broad array of secondary metabolites, through a cascade of biochemical processes. Representative bacterial endophytes, their plant sources, and corresponding target pathogens are summarized in Table 1. Purifying such metabolites along with discovery of new ones without losing their antimicrobial potential serves as a sustainable alternative to healthcare sectors to fight high priority pathogens and combating the ever-rising AMR and MDR trends. The compilation of data in Table 1 clearly underlines the limitation of ongoing research in the field where emphasis is placed on crude or semi-purified compounds rather than focusing on a single lead metabolite. The MIC values reported provide evidence for antimicrobial activity, but the translational potential often remains restricted. This implies that a single metabolite-based approach is essential and necessary for drug development as it can then focus on the structure of metabolite and predict and validate the mechanism of action. Such specific approaches are critical in drug development pipelines while coming up with a natural antimicrobial molecule from endophytes.

**TABLE 1:** Endophytic origins of bioactive metabolites targeting human pathogens.

Plant source	Endophyte	Target pathogens	MIC (µg/mL)	Metabolite	Chemical class	Known or hypothesized mechanism of action	Solvents used for extraction	References
*Boswellia sacra* (leaves and stem)	*Bacillus subtilis*	*Escherichia coli*	-	cyclo-(L-pro-L-val); cyclo-(L-pro-L-phe)	Cyclic dipeptides (diketopiperazine)	Suppression of β-lactam biosynthesis related genes (ACVS and AADAT)	Ethyl acetate	Numan et al. (2022)
*Hieracium canadense* (roots)	*Micromonospora chokoriensis*	Methicillin resistant *Staphylococcus aureus*	8	Bagremycin A (BagA)	Phenolic ester	Docking: BagA inhibits thymidylate kinase (TMK)	-	Tanvir et al. (2024)
		*Staphylococcus aureus*						
		*Candida tropicalis*						
		*Enterococcus* sp	16					
		*Bacillus subtilis*						
		*Saccharomyces cerevisiae*						
		*Chlorella vulgaris*						
*Rauvolfia serpentina* (leaves)	*Bacillus mojavensis*	*Escherichia coli*	100	Reserpine	Indole alkaloid	-	Ethyl acetate	Lata and Gond et al. (2025)
		*Proteus vulgaris*	100					
		*Citrobacter freundii*	100					
		*Morganella morganii*	200					
		*Citrobacter koserii*	100					
		*Enterococcus faecalis*	100					
		Methicillin resistant *Staphylococcus aureus*	<100					
		*Klebsiella pneumoniae*	100					
		*Shigella boydii*	<100					
		*Pseudomonas aeruginosa*	100					
	*Bacillus wiedmannii*	*Escherichia coli*	800	Reserpine	Indole alkaloid	-		
		*Proteus vulgaris*	400					
		*Citrobacter freundii*	800					
		*Morganella morganii*	100					
		*Citrobacter koserii*	200					
	​	*Enterococcus faecalis*	<100	​	​	​	​	​
		Methicillin resistant *Staphylococcus aureus*	3,200					
		*Klebsiella pneumoniae*	200					
		*Shigella boydii*	100					
		*Pseudomonas aeruginosa*	1,600					
*Archidendron pauciflorum* (leaf, stem, root)	*Priestia* (*Bacillus) megaterium*	*Escherichia coli*	62.50	Baptifoline; dehydromorroniaglycone; sophoramine; 1,4-dihydroxy-2-methyl-anthraquinone	Alkaloids, iridoid derivates, and anthraquinones	Mechanism of action is not fully elucidated, but inhibition of biofilm formation is demonstrated	Ethyl acetate	Priyanto et al. (2023)
		*Pseudomonas aeruginosa*	31.25					
		*Bacillus subtilis*	62.50					
		*Klebsiella pneumoniae*	1,000					
	*Bacillus amyloliquefaciens*	*Escherichia coli*	31.25	Baptifoline; dehydromorroniaglycone; sophoramine; paenilamicin A1	Alkaloids, iridoid derivatives, and peptide antibiotics (non ribosomal peptides)			
		*Pseudomonas aeruginosa*	7.81					
		*Bacillus subtilis*	31.25					
		*Klebsiella pneumoniae*	1,000					
	*Bacillus pseudomycoides*	*Escherichia coli*	62.25	Baptifoline; dehydromorroniaglycone; isoleucinopine; anisalacetone	Alkaloids, iridoid derivatives and phenylpropanoids			
		*Pseudomonas aeruginosa*	62.25					
		*Bacillus subtilis*	125					
		*Klebsiella pneumoniae*	62.25					
	*Bacillus velezensis*	*Escherichia coli*	15.62	Baptifoline; dehydromorroniaglycone; isoleucinopine; paenilamicin A1	Alkaloids, iridoid derivatives, amino acid – derived metabolites, and peptide antibiotics			
		*Pseudomonas aeruginosa*	7.81					
		*Bacillus subtilis*	31.25					
		*Klebsiella pneumoniae*	250					
*Gracilaria edulis*	*Bacillus subtilis*	*Bacillus cereus*	Not determined (only % inhibition at 50–200 μg/mL)	Pyrrolo [1,2-α]pyrazine-1,4-dione, hexahydro-3-(2-methylpropyl)	Diketopiperazine alkaloid	Docking: β-lactamase inhibitor	Chloroform	Manam et al. (2025a)
		*Pseudomonas aeruginosa*						
		*Escherichia coli*						
*Solanum mauritianum* (fruits, stems, leaves)	*Pantoea ananatis*	*Bacillus cereus*	125	Dubinidine; genipin; xanthosine; droserone; 4-methyl-5-(2′-hydroxyethyl)-thiazole; anatabine; khellin; (R)-columbianetin; 1-methyluric acid; sinapyl alcohol; eupaformosanin; fulvoplumierin; (−)-Laudanidine; asebogenin; picropodophyllin; naringenin; borrerine; protodioscin	Alkaloids, quinones, phenylpropanoids, flavonoids, terpenoids, coumarins, lignans, iridoids, and steroidal saponins	-	Ethyl acetate	Ogofure and Green (2025)
		*Bacillus subtilis*	250					
		*Enterobacter aerogenes*	4,000					
		*Escherichia coli*	500					
		*Klebsiella pneumoniae*	2000					
		*Mycobacterium marinum*	125					
		*Mycobacterium smegmatis*	125					
		*Proteus vulgaris*	500					
		*Pseudomonas aeruginosa*	1,000					
		*Staphylococcus aureus*	63					
		*Staphylococcus epidermidis*	125					
	*Bacillus licheniformis*	*Bacillus cereus*	250	Dubinidine; genipin; xanthosine; droserone; 4-methyl-5-(2′-hydroxyethyl)-thiazole; anatabine; khellin; (R)-columbianetin; sinapyl alcohol; eupaformosanin; fulvoplumierin; (−)-Laudanidine; asebogenin; picropodophyllin; protodioscin; visnagin; arborinine; rosinidin; vasicinone; isolobinine; tuliposide A; dauricine; cannabielsoin; cassine; neoquassin	Alkaloids, quinones, flavonoids, terpenoids, coumarins, lignans, iridoids, glycosides, steroidal saponins, and cannabinoids	-		
		*Bacillus subtilis*	250					
		*Enterobacter aerogenes*	1,000					
		*Escherichia coli*	1,000					
		*Klebsiella pneumoniae*	2000					
		*Mycobacterium marinum*	500					
		*Mycobacterium smegmatis*	500					
		*Proteus vulgaris*	1,000					
		*Pseudomonas aeruginosa*	1,000					
		*Staphylococcus aureus*	63					
		*Staphylococcus epidermidis*	125					
​	*Arthrobacter* sp.	*Bacillus cereus*	63	Dubinidine; genipin; xanthosine; droserone; 4-methyl-5-(2′-hydroxyethyl)-thiazole; anatabine; visnagin; khellin; (R)-columbianetin; 1-methyluric acid; sinapyl alcohol; eupaformosanin; fulvoplumierin; sophoraisoflavanone A; casimiroin; rosinidin; vasicinone; mallotophenone; asebogenin; picropodophyllin; tuliposide A; cannabielsoin; protodioscin; neoquassin	Alkaloids, quinones, flavonoids, coumarins, phenylpropanoids, terpenoids, lignans, iridoids, glycosides, steroidal saponins, cannabinoids, and quassinoids	-	​	​
		*Bacillus subtilis*	125					
		*Enterobacter aerogenes*	500					
		*Escherichia coli*	250					
		*Klebsiella pneumoniae*	500					
		*Mycobacterium marinum*	250					
		*Mycobacterium smegmatis*	1,000					
		*Proteus vulgaris*	500					
		*Pseudomonas aeruginosa*	250					
		*Staphylococcus aureus*	63					
		*Staphylococcus epidermidis*	125					
*Ocimum sanctum* (stem, leaves, petiole)	*Enterobacter cloacae*	*Pseudomonas aeruginosa*	6,250	Carbamic acid, butyl-, ethyl ester; benzeneacetic acid, alpha.-oxo-, methyl; benzene, (1-methoxyethyl)-; 2-naphthalenol; Colchicine, (+)-; diethyl phthalate; silane, tetramethyl; benzene, 1-methyl-2-(1-methylethyl)-; 4-isopropyltoluene; octachlorobiphenyl; 2,4,6-trihydroxybenzaldehyde; Di-N-butylphthalate; silane, diethyl methyl; BIS(2-Ethylhexyl) phthalate	Phthalate esters, aromatic hydrocarbons, phenolic compounds, organosilicon compounds, alkaloids, and aromatic ester derivatives	Mechanism of action not fully elucidated; BLIS (bacteriocin like inhibition studies) suggests bacteriocin like activity	Butanone: Ethyl acetate: ethanol: Water (3:5:1:1)	Panigrahi and Rath, 2021; Panigrahi et al., 2020
		*Escherichia coli*	6,250					
		*Staphylococcus aureus*	1,560					
		*Klebsiella pneumoniae*	3,120					
		*Salmonella enteric*	6,250					
		*Bacillus subtilis*	390					
		*Xanthomonas oryzae*	3,120					
*Solanum lycopersicum* (roots)	*Streptomyces sp*	*Pseudomonas aeruginosa*	Ranges from 10 to 15 μg/mL	Kendomycin	Polyketide antibiotic	-	Ethyl acetate	Veilumuthu et al. (2024)
		*Escherichia coli*						
		Methicillin resistant *Staphylococcus aureus*						
		*Vancomycin resistant enterococci*						
		*Klebsiella pneumoniae*						
		*Escherichia coli*						
*Crinum macowanii* (leaves)	*Bacillus safensis*	*Bacillus cereus*	>16,000	Lycorine; angustine; 3-O-methyl epimacowine; crinamidine; vasicinol; powelline	Alkaloids	-	Ethyl acetate	Sebola et al., 2020
		*Bacillus subtilis*	125					
		*Staphylococcus epidermis*	2000					
		*Staphylococcus aureus*	>16,000					
		*Mycobacterium smegmatis*	4,000					
		*Mycobacterium marinum*	500					
		*Enterobacter aerogenes*	16,000					
		*Escherichia coli*	250					
		*Klebsiella pneumoniae*	2000					
		*Proteus vulgaris*	4,000					
		*Pseudomonas aeruginosa*	>16,000					
	*Pseudomonas cichorii*	*Bacillus cereus*	8,000	Lycorine; angustine; crinamidine; vasicinol; aulicine; powelline	Alkaloids	-		
		*Bacillus subtilis*	250					
		*Staphylococcus epidermis*	16,000					
		*Staphylococcus aureus*	1,000					
		*Mycobacterium smegmatis*	125					
		*Mycobacterium marinum*	8,000					
		*Enterobacter aerogenes*	4,000					
		*Escherichia coli*	1,000					
		*Klebsiella pneumoniae*	500					
		*Proteus vulgaris*	1,000					
		*Pseudomonas aeruginosa*	125					
	*Arthrobacter pascens*	*Bacillus cereus*	2000	Lycorine; angustine; crinamidine; vasicinol; powelline; brefeldin A	Alkaloids, and polyketides	-		
		*Bacillus subtilis*	62.5					
		*Staphylococcus epidermis*	4,000					
		*Staphylococcus aureus*	1,000					
		*Mycobacterium smegmatis*	16,000					
		*Mycobacterium marinum*	>16,000					
​	​	*Enterobacter aerogenes*	1,000	​	​	​	​	​
		*Escherichia coli*	500					
		*Klebsiella pneumoniae*	>16,000					
		*Proteus vulgaris*	2000					
		*Pseudomonas aeruginosa*	4,000					

The biosynthesis of these small molecules is governed by specialized enzymatic systems such as polyketide synthases (PKS) and non-ribosomal peptide synthases (NRPS), which convert primary precursors into chemically diverse, bioactive scaffolds (Nimbulkar et al., 2025). Advances in genomics and bioinformatics have facilitated the identification of biosynthetic gene clusters (BGCs) responsible for these pathways. For instance, *Bacillus cereus* harbors clusters encoding bacillibactin, zwitterion, and petrobactin, while *Streptomyces* species possess hybrid PKS–NRPS systems producing multiple antimicrobials (Nimbulkar et al., 2025; Singh et al., 2025).

**FIGURE 1:**
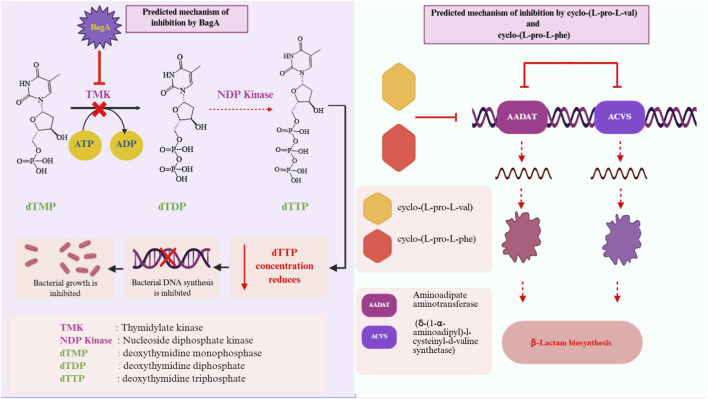
Endophytic metabolites and their probable mechanisms of action against human pathogens. Created in BioRender. Osborne, J. (2026) https://BioRender.com/qeq3y7h.

These metabolites act through diverse mechanisms, including disruption of membrane integrity (Sharma et al., 2022; Jasim et al., 2016), inhibition of cell wall synthesis (Ramírez-Rendó, 2023; Castiglione et al., 2007), and interference with nucleic acid or protein synthesis (Manzano et al., 2025; Antony et al., 2024). Predicted mechanism of action of cyclo-(L-pro-L-val), cyclo-(L-pro-L-phe) and Bagremycin A (BagA) (Tanvir et al., 2024; Numan et al., 2022; Keating et al., 2012; Venugopala et al., 2020) listed in Table 1 is presented in Figure 1.

## 3 Synthesizing bioactive metabolites as API from endophytes; a strategic framework for upstream bioprocess development

Upstream processes encompass all the steps involved in developing and optimizing microbial systems to produce desired metabolites and products (Liu et al., 2025). Classical approaches to API production from bacterial endophytes focus only on culturable microorganisms, while the major bottleneck regarding ‘Great Plate Count Anomaly’ persists leaving behind unculturable microorganisms under laboratory conditions (De la Vega-Camarillo et al., 2026). Reports indicate that only approximately 0.1%–10% of microorganisms are retrievable in culturable form (Khan and Saha, 2026). Consequently, reliance on conventional agar plate-based methods for endophytic bacterial isolation may lead to the loss of potentially valuable and unexplored bioactive compounds from unculturable populations. Modern methodologies incorporating metagenomics and methods like iChip (isolation chips) can help solve this problem and expand the study further to underexplored fields.

### 3.1 Classical culture dependent upstream processing of endophytic bacterial metabolites

**FIGURE 2:**
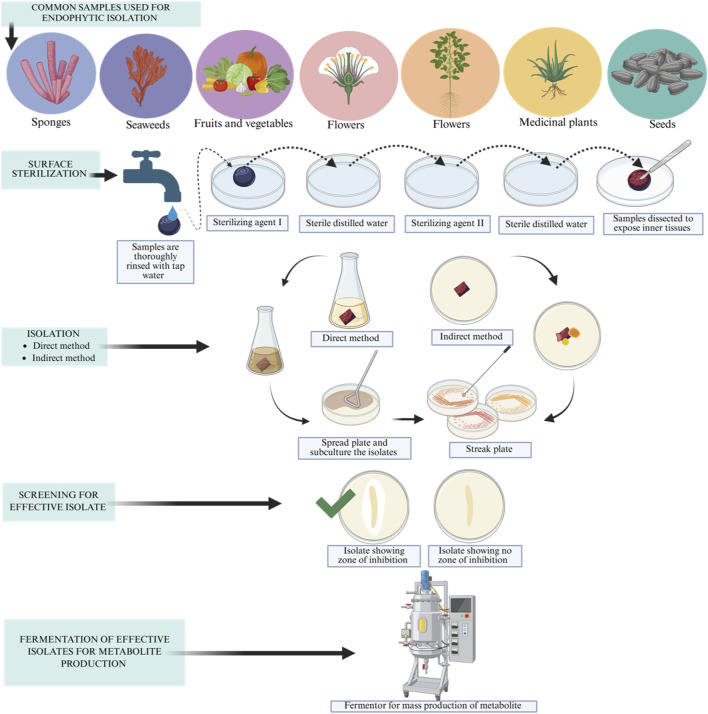
Schematic overview of upstream processes in endophytic research. Created in BioRender. Osborne, J. (2026) https://BioRender.com/x0xnmse.

Culture dependent upstream processes for endophytic bacterial metabolite production comprise of a series of steps initiating with the isolation of endophytic bacteria, followed by screening assays to confirm the desired bioactivity, and fermentation for mass multiplication of the metabolites for various downstream processes (Yasu, 2024), a schematic overview of which is represented in Figure 2. Culture dependent upstream focuses primarily on bacteria that are readily cultivable under laboratory conditions. A key limitation of relying exclusively on culturable bacteria is an increased likelihood of rediscovery of previously characterized metabolites thereby blocking access to novel bioactive compounds (Lasch et al., 2026). The efficiency of upstream process is dependent on multiple critical parameters. Optimization of culture conditions in terms of nutrient composition, temperature, pH, and salinity are paramount for enhancing metabolite production. Peng et al. (2024), indicated that accurate manipulations of the concentrations of nutrients led to significant increase in bacteriocin (desired product) yields and ensured stability in the ranges of 30 °C–40 °C and pH 5-7 implying the importance of providing optimal conditions for improved stability and productivity.

#### 3.1.1 Isolation of culturable endophytic bacteria: resolving the tight spot of epiphytic bias

Isolation of endophytic bacteria begins with surface sterilization, a crucial step that eliminates epiphytic microorganisms while preserving internal endophytes. Commonly used plant tissues include roots (Nwajiobi et al., 2025), fruits (Khuder and Mohammed, 2025) and leaves (Preethi and Umesha, 2025). The efficacy of the method basically relies on the type of sample used and concentration and time of exposure to sterilizing agents (Srivastava et al., 2024; Sahu et al., 2022). Several investigations therefore focus on optimization of such parameters to effectively remove the unwanted epiphytes and retain the endophytic populations (Srivastava et al., 2024). Sterility check is of prime importance in endophytic research which is basically performed by rolling the surface sterilized samples and plating the sterilizing agents on nutrient agar to rule out the chances of misidentifying an epiphyte as an endophyte (Manam et al., 2025b). This step is of prime importance in avoiding any bias with the isolation of endophytes as this helps in excluding the epiphytic contaminants on sterility plates from the impregnation plates. However, application of only this method may not be sufficient to rule out completely the chances of epiphytic contamination particularly if molecular studies are involved. It has been reported that commonly used sterilizing agents may not be capable of degrading the DNA of epiphytic microorganisms present which may later mislead the study and contribute to epiphytic bias (Burgdorf et al., 2014). Therefore, followed by surface sterilization, the final wash distilled water should be subjected to PCR amplification using universal primers of 16S rRNA genes so as to compare and eliminate the epiphyte from endophyte (Dendooven et al., 2024). As summarized in Table 2, procedures vary with plant species, tissue type and microbial richness, yet certain patterns remain uniform. Treatment with ethanol and sodium hypochlorite (NaOCl) in a sequence remains the standard, with ethanol (70%–90%, 30 s - 2 min) for primary decontamination, followed by NaOCl (2%–6%, 2–10 min) for complete removal of epiphytes.

**TABLE 2:** Various surface sterilizing agents used for the isolation of endophytic organisms from diverse plant hosts are provided below.

Plant source	Plant part used	Sterilizing agent	Time of exposure	Sterility validation method	References
*Moringa oleifera*	Leaves, roots, dehusked seeds	70% ethanol	2 min	Final rinse plating	Gul et al. (2025)
		2.5% sodium hypochlorite	4–5 min		
Sorghum Sudan grass	Seeds	4% sodium hypochlorite	10 min	Final rinse plating	Kumar K. et al. (2025)
		70% ethanol	30 s		
*Cynodon dactylon*	Whole plant	70% ethanol	2 min	Final rinse plating	Kumar H. et al. (2025)
		4% sodium hypochlorite	1 min		
*Styrax paralleloneurus*	Stems	75% ethanol	1 min	Final rinse plating	Susilowati et al. (2025)
		Sodium hypochlorite	5 min		
		75% ethanol	30 s		
Sea grass	Whole plant	90% ethanol	10 s	Final rinse plating; uncut explant imprinting	Kandasamy et al. (2025)
		4% sodium hypochlorite	20 s		
		75% ethanol	30 s		
*Hibiscus cannabinus,* Zea mays	Roots	95% ethanol	1 min	Final rinse plating	Nwajiobi et al. (2025)
		3% sodium hypochlorite	3 min		
		70% ethanol	30 s		
*Zanthoxylum dissitum*	Stems	75% ethanol	2 min	Final rinse plating	Fei et al. (2025)
		3% sodium hypochlorite	2 min		
*Sphagnum palustre*	Whole plant	75% ethanol	30 s	Final rinse plating	Wang et al. (2025)
		Hydrogen peroxide	6 min		
*Dioscorea opposita*	Leaves	70% ethanol	30 s	Final rinse plating	Li et al. (2025)
		0.1% mercuric chloride solution	5 min		
*Endostemon obtusifolius*	Roots; leaves	Tween 20 (3 drops)	1 min	Final rinse plating; explant imprinting	Ogbe et al. (2023)
		0.1% carbendazim	20 min		
		70% ethanol	60 s		
		2% sodium hypochlorite	60 s		
		70% ethanol	30 s		
*Thymes kotschyanus, Allium hooshidaryae, Cerasus macrocarpa*	Roots, leaves, stems	1% tween 20	5 min	Final rinse plating; explant imprinting	Delbari et al. (2023)
		70% ethanol	5 min		
		6% sodium hypochlorite	5 min		
		10% sodium bicarbonate	10 min		
*Abies religiosa*	Roots, aerial parts of shoot	2.5% commercial bleach	5 min; 15 min (root)	PCR based sterility validation; (final rinse water used as template for 16S rRNA PCR)	Dendooven et al. (2024)
		70% ethanol	5 min		

Hydrogen peroxide, mercuric chloride, carbendazim, sodium bicarbonate, and surfactants (Tween-20), in a combination with ethanol and/or NaOCl are also used, although their use is comparatively less common (Mahdi et al., 2022). Harder tissues like seeds and roots require stronger and prolonged treatments whereas softer tissues like nodules require only mild conditions. Multiple rinses with sterile distilled water are advised after every treatment to ensure proper endophyte recovery and remove residual sterilant toxicity (Mahdi et al., 2022; Susilowati et al., 2025). Overall, an optimized sequential process with a pre-treatment with ethanol, a disinfection with NaOCl and a final ethanol rinse, has proved to be the most effective method for isolating endophytes from various plant hosts.

Surface sterilization is followed by impregnation and/or enrichment methods for isolation of endophytes (Dey et al., 2023; Agarwal and Sheikh, 2025; Ramalashmi et al., 2018). Sterilized tissues are excised under aseptic conditions into 1mm-1 cm accordingly (Biswas et al., 2023; Singh et al., 2022) and placed on appropriate media by impregnation method whereas sterilized intact or homogenized tissues are incubated in broth with an agitation of 120 rpm to support microbial release and growth, which is then followed by serial dilution and plating to obtain morphologically different distinct colonies in enrichment method (Buragohain et al., 2023). The selection of media is crucial for isolating diverse range of endophytes and is tailored to suit the ecological niche of the host. For example, terrestrial plant samples and tissues are processed on general purpose media such as Nutrient agar (Pang et al., 2022), Luria Bertani agar (Agarwal and Sheikh, 2025) or Tryptic Soy Agar (Munif et al., 2025) while working with marine samples require specialized media like Zobel Marine Agar (Manam et al., 2025a) or general-purpose media supplemented with sea salts while sterility plates are always prepared using general purpose media to permit the growth of diverse bacterial contaminants to ensure elimination of epiphytic and environmental contaminants prior to endophyte purification.

#### 3.1.2 Effective screening of endophytic bacteria exhibiting antagonistic activity against selected human pathogens

Systematic screening is crucial in endophytic research to identify the most potent isolate showing inhibitory activity against pathogens of interest and thus helps in narrowing down the number of isolates that must be studied extensively. Also, performing an antibacterial assay at each stage of compound purification ensures that the purification and fractionation steps followed are progressing in the correct trajectory. Table 3 summarizes the commonly adopted methods and their respective advantages and limitations that must be evaluated carefully to the study objective.

**TABLE 3:** Overview of widely used screening techniques with their advantages and limitations.

Assay method	McFarland no. maintained	Incubation condition	Evaluation methods	Advantages	Limitations	References
Disc diffusion assay	0.5	37 °C overnight	Zone of inhibition in mm	Cost- effective, widely used and standardized for various drug- organism pairs	Non- quantitative	Hossain et al. (2024),Rustini et al. (2023); Gajic et al. (2022)
Agar well diffusion assay	0.5	37 °C for 48 h	Zone of inhibition in mm	Low technical demand; limited equipment requirement	Non- quantitative	Myo et al. (2020); Ojha and Jangid (2025)
Double layer agar diffusion assay	0.5	37 °C for 24 h	Zone of inhibition in mm	Direct culture use; sustained metabolite release	Non- quantitative	Khairani et al. (2025)
Cross streak assay	0.3	37 °C for 24 h	Zone of inhibition in mm	Direct visual readout, allows multiple test organisms per plate	Non- quantitative, diffusion dependent, variability in inhibition zone size	Hossain et al. (2024); Buragohain et al. (2023)
Agar dilution method	0.5	37 °C	MIC values	-	-	Chiu et al. (2021)
Broth macro dilution method	1–2 mL; 0.5	36 °C ± 2 °C (6,12, 18, 24 h)	MIC values	Quantitative assessment of MIC values, standardized by CLSI/EUCAST	High reagent use; single antimicrobial per assay	Hossain et al. (2024); Basit et al. (2025)
Broth microdilution method	100–200 µL	37 °C overnight, (18–24 h)	MIC values	Simultaneous multi-drug testing; automation- compatible	Solvent interference: Hydrophobic compounds may precipitate; multiple dilutions required	Hossain et al. (2024); Fanoro and Oluwafemi (2025)
Antimicrobial gradient method	0.5	16–24 h at 35 °C ± 2 °C (aerobic conditions)	MIC read at ellipse-scale intersection; automated readers available (ADAGIO)	Quantitative; commercially available for bacteria and fungi	Results influenced by agar composition; limited antibiotic capacity per plate	Gajic et al. (2022)

#### 3.1.3 Identification and molecular characterization of the effective endophytic bacteria

Antibacterial screening preliminarily confirms the effectiveness of the isolates. This should be followed by molecular identification of the effective isolate for further analysis. DNA extraction is the first step involved in molecular workflow and is usually carried out using commercial DNA extraction kits or by Cetyl Trimethyl Ammonium Bromide (CTAB) method. Purity of DNA is crucial for successful downstream molecular applications. Budi and Khoir, (2026) both the DNA isolation methods and reported that CTAB method yielded a DNA purity of 1.83 whereas commercial kit yielded a purity of 2.04 indicating higher efficiency and lower susceptibility to contamination in commercial kits. Followed by genomic DNA isolation, PCR amplification of 16S rRNA gene should be performed using the universal primers 27F and 1492R. Amplification can be confirmed by agarose gel electrophoresis. This will be followed by sequencing for molecular identification of the isolate.

Sanger sequencing of the 16S rRNA gene is the most widely employed method for molecular identification of bacterial isolates due to its highly conserved nature within bacteria and its high accuracy. However, as it analyses only a short, sequenced region, it is often insufficient for distinguishing closely related bacteria. Therefore, genus and species level identification often becomes challenging (Campodónico et al., 2025). Furthermore, chimera formation and variation in PCR amplification can also affect the efficiency of 16S rRNA gene Sanger sequencing (Ivens et al., 2025). Sanger sequencing is often too slow and expensive therefore, next-generation sequencing approaches like Illumina sequencing were introduced. Illumina sequencing generates millions of short, paired reads and error rate is less than 0.1%. It mainly aims to target hypervariable regions of 16S rRNA gene enabling genus level resolution. Species level resolution is very low here (Macip et al., 2025).

Taxonomic resolution can be improved by sequencing the full length 16S rRNA gene. To rectify this challenge with short read sequencing, researchers have developed long read nanopore sequencing methods. The latest version of Long-read nanopore sequencing, the R10 sequencing chemistry reads both strands of same DNA in forward and reverse thus reduces error. Furthermore, it addresses errors associated with homopolymers and produces high quality reads (>99% accuracy) (Campodónico et al., 2025; Ivens et al., 2025). Sequencing data obtained can be analyzed using BLAST-N available in NCBI database to determine sequence similarity and closely related organisms. Phylogenetic trees can be constructed using software like ClustalW and MEGA7 to determine evolutionary relationships among isolates (Budi and Khoir, 2026; Tang et al., 2026). Finally, the sequence should be subjected to NCBI to acquire the sequence accession numbers (Tang et al., 2026).

#### 3.1.4 Metabolite production by fermenting effective microbial endophytes

Upon identification of effective endophyte capable of target action, the production of metabolites is enhanced for further screening and identification through fermentation. Lab scale fermentation is later scaled up to bioreactor levels for mass production of metabolites. Process of fermentation enables the conversion of organic substrates into various bioactive molecules like antibiotics and enzymes (Yadav et al., 2024). Studies by Manam et al. (2025a), reported the production of effective secondary metabolites from endophyte of *Gracilaria edulis*, and investigations by Lata and Gond et al. (2025) demonstrates the achievement of enhanced antibacterial yields through optimized bacterial fermentation, thereby confirming the importance of fermentation in bridging the gap between lab-scale and industrial scale metabolite recovery. More of the recent studies focusses on optimization of fermentation parameters like nutrient sources (carbon, nitrogen), incubation period, temperature, pH etc., to improve yield and product quality. Several one-factor-at-a-time (OFAT) approaches for optimization are being performed widely and by combining advanced statistical and computational tools like Response Surface Methodology (RSM) (Hu et al., 2024; Hellany et al., 2024) has contributed significantly to the product yield. Therefore, it is crucial to formulate a method to identify the optimal conditions of effective isolate and validate the same experimentally to yield a reproducible result. Researchers often initiate their media optimization via OFAT and minimize the number of factors with the help of Plackett-Burman and optimal conditions and interactive nature is studied using RSM (Bencherif and Hassaine, 2025). The use of these statistical and computational tools will help in narrowing down the factors and thus require minimal efforts and maximum yield and saving time in optimization studies.

However, precision fermentation using optimized media may not always result in increased yield. In certain cases, bioactivity may not be observed even after optimization which may be associated with cryptic BGCs. Studies have reported that certain secondary metabolites are produced because of the interaction between plants and microbes (Petijová et al., 2024). Upon isolation and cultivation under laboratory conditions, these interactions and environmental cues which triggers cryptic BGC activation are absent, which may prevent expression of certain BGCs leaving them silent. Furthermore, repeated subculturing and prolonged maintenance under laboratory conditions may lead to loss of metabolite-producing efficiency by silencing BGCs encoding the target genes (Zakariyah et al., 2024). These cryptic pathways are to be activated and expressed for effective retrieval of novel metabolites of bioactive interest. Co-cultivation of microorganisms has been established as an important strategy for activating the cryptic BGCs (Zakariyah et al., 2024), as evidenced by the renowned discovery of penicillin, where contamination of *Staphylococcus* with *Penicillium notatum* resulted in penicillin production (Fleming, 1929). This supports to the idea of co-cultivation and has been reported to transcriptionally trigger silent BGCs, thereby inducing the expression of bioactive metabolites.

One Strain Many Compounds (OSMAC) is a method of activating silent BGCs by leaning on to the concept that a single microorganism can produce different types and sets of metabolites when exposed to varying conditions. By altering factors like pH, temperature, nutrients, and aeration diverse range of biosynthetic pathways can be activated producing variety of bioactive metabolites (Romano et al., 2018). Chemical elicitors are often reported to have the ability to activate silent biosynthetic pathways and are also known to accelerate the yield to several folds. Methods like HiTES (High-Throughput Elicitor Screening) are based of fluorescent assay for identifying the elicitors that activate the desired silent BGCs (Tyurin et al., 2018). These chemical elicitor molecules act on silent BGCs by inducing epigenetic modifications by relaxing the heterochromatin and converting it to euchromatin (Sharma et al., 2024). This is often achieved by inhibiting DNA methylation and histone deacetylation (Bharatiya et al., 2021). Histone deacetylation is inhibited usually by elicitors like SAHA (Suberanilohydroxamine acid) and sodium butyrate whereas DNA methylation is inhibited by 5-azacytidine molecule (Anjum and Xuewei, 2022) Liao et al. (2025) reported that cultivation of isolate with SAHA resulted in production of undescribed sesquiterpene molecules whereas Terkar et al., 2026 explicitly states that Azacitidine and sodium butyrate when exposed to two different endophytes resulted in production of 9–11 silent metabolites in each case.

Mass production of lead metabolites can be achieved through a scale-up from shake-flasks to bioreactors. This scale-up introduces complexity and additional parameters involved such as dissolved oxygen, agitation speed, and aeration which enhances productivity (Boruta et al., 2023).

Fermentation, when becomes scaled up to industrial stage, employs different types of bioreactors mostly classified as batch, fed batch and continuous systems, decided based on primary aim of the study. These reactors witness bioreactions under dynamic or static conditions and can operate in solid or liquid states (Xie et al., 2022 ). Table 4 provides an overview of bioreactors and culture conditions employed for large scale production of metabolites.

**TABLE 4:** Overview of bacterial strains, bioreactor systems, culture conditions and metabolites used in scale up studies.

Producer organism	Type of bioreactor	Model	Working volume	Culture conditions	Metabolites/Enzymes produced	References
*Pediococcus pentosaceus*	Stirred tank bioreactor (batch)	BIOSTAT® B (sartorius, goettingen, Germany)	1.5 L	Speed of stirring: 200 rpm; temperature: 30 °C; nitrogen was purged before inoculation to ensure anaerobic condition	BLIS (bacteriocin like inhibitory substance)	De Azevedo et al. (2019)
*Lactobacillus plantarum*	Stirred tank bioreactor	Biostat B plus (sartorius, gottingen, Germany)	1 L (0.9 L medium +0.1 L inoculum)	Speed of stirring: 100 rpm, temperature: 30 °C	Bacteriocin	Sabo et al. (2019)
*Streptomyces kanasenisi*	Stirred tank bioreactor (bench-scale fermentor)	GBCN-5C (5 L glass); GUJS-15; GUJS-70; GJ-500 (stainless steel)zhenjiang east biotech equipment and technology co., Ltd., zhenjiang, zhenjiang, China	3.5 L (bench - scale); 10 L, 50 L, 350 L (pilot-scale)	Agitation: by two flat blades in 5 L, two six- flat blade in pilot scale; aeration: spider sparger; temperature: maintained by heating-cooling system; DO monitored	Glycoprotein GP-1	Zhou et al. (2018)
*Bacillus velezensis* AmoreLumina	Stirred tank bioreactor (benchtop); batch and fed batch	MARADO; CNS	3 L (in 5 L fermentor)	Agitation: 400 rpm (300–600 rpm tested for optimization); Aeration: 1vvm; impeller: disc turbine type (2 units); sparger: ring type	Moranoline	Ju et al. (2025)
*Streptococcus zooepidemicus*	Stirred tank bioreactor (batch)	SGB-10l, changzhou sungod nio-technology and engineering eqipment CO., Ltd., Jiangsu, China	7 L (in 10 L fermentor)	Agitation: 600 rpm; Aeration: 1 vvm; temperature: 37 °C	Hyaluronic acid	Liu et al. (2018)
*Bacillus subtilis*	Fed-batch bioreactor	BIOSTAT® B (sartorius, Germany)	1.2 L in 2 L benchtop bioreactor	Temperature: 30 °C Aeration: 1 vvm Feeding: 500 g/L sucrose was supplemented on residual sugar concentration drop to 10 g/L	2,3-Butanediol	Suttikul et al. (2022)
*Streptomyces atratus*	Stirred tank bioreactor	Shanghai Guoqiang biochemical engineering equipment co., Ltd., Shanghai, China	2.7 L in 5 L bioreactor	Temperature: 26 °C Aeration: 1–2 vvm Agitation: 200–600 rpm (based on DO level)	Ilamycins	Jiang et al. (2023)

### 3.2 Modern culture-independent downstream processing of endophytic bacterial metabolites

Classical agar-based techniques employed for the isolation of endophytes in metabolite discovery are often inadequate for the identification of novel bioactive compounds, as a significant fraction of microorganisms remain unculturable under conventional lab-based conditions. To overcome this limitation modern approaches incorporating metagenomics, and *in-situ* culturing methods like plate-wash PCR, iChip methods are extensively used.

#### 3.2.1 Metagenomics as a tool for exploring chemical dark matter

Metagenomics is the study of identification of both culturable and unculturable microorganisms after extraction of DNA from a sample and sequencing using appropriate primers and it provides data for accurate understanding of the functions and diversity (Hong et al., 2019). By understanding the functional capabilities of the entire endophytic community, access to chemical dark matter and its extraction can be directed according to the desired target, which is otherwise not possible through classical culture dependent approaches. Through classical culture-based methods highly expressed biosynthetic gene clusters (BGC) can be studied whereas in metagenomics the cryptic BGCs are also accessible opening doors to chemical dark matter (Lasch et al., 2026). Metagenomics mainly is divided into amplicon and shotgun metagenomics in which the former one deals with microbial diversity whereas the latter deals with microbial diversity and their functional roles. Following sample collection and DNA extraction, sequencing, bioinformatic analysis and data visualization are performed to obtain comprehensive metagenomic data (Nam et al., 2023).

Shotgun metagenomic sequencing is of prime importance in metabolite research as it provides more data about functional roles of genes (Fadiji et al., 2021). Once the targeted cryptic BGC is identified, a successful heterologous gene expression will result in expression of these genes and the production of respective bioactive molecules in a culturable host (Lasch et al., 2026). Although BGCs provide critical insights into microbial metabolite production, BGC mining from large metagenomic datasets remains a significant challenge. The exploration of novel and previously uncharacterized metabolites depends mainly on accurate identification of complete BGCs. Conventional short read sequencing methods commonly involved often fail to detect all BGCs present within metagenomic data. This challenge is addressed by PacBio’s High Fidelity (HiFi) technology for sequencing to detect and recover repetitive and long BGCs like non-ribosomal peptide synthetase (NRPS) and polyketide synthase (PKS) which are difficult to identify using short-read sequencing approaches (Yadav and Subramanian, 2024).

#### 3.2.2 *In-situ* cultivation methods for accessing chemical dark matter

Unculturable microbes were always a greater importance for discussion. *In-situ* cultivation approaches such as diffusion chambers and isolation chips (iChips) have shown significant potential in facilitating the growth of previously unculturable microorganisms. Diffusion chambers are positioned within a native environmental niche, allowing the diffusion of essential nutrients and signaling molecules required for microbial growth and thereby promoting the cultivation of otherwise unculturable bacteria. Diffusion chambers will allow the microbes growing to utilize various biotic and abiotic environmental cues thereby mimicking the external environment inside chambers. Therefore, the diversity of microbes growing inside diffusion chambers will be more compared to traditional agar plate methods (Kumar, 2026).

iChip is also another effective *in-situ* cultivation approach and is a modification of diffusion chambers. iChips are made of hundreds of wells that act as diffusion chambers. Microbial entry from each diffusion chambers are restricted with the help of membranes with pore size smaller than that of bacteria. The iChips are transferred to the sample collected region so that the environmental niche is same and the nutrients and conditions that the microbes require to grow are satisfied till microcolonies appear (Nazipi Bushi et al., 2025). In context of endophytic bioactive metabolite discovery, limited studies are only conducted employing *in-situ* cultivation methods.

## 4 Fermented metabolites to bioproduct development: a targeted approach to single metabolite detection

**FIGURE 3:**
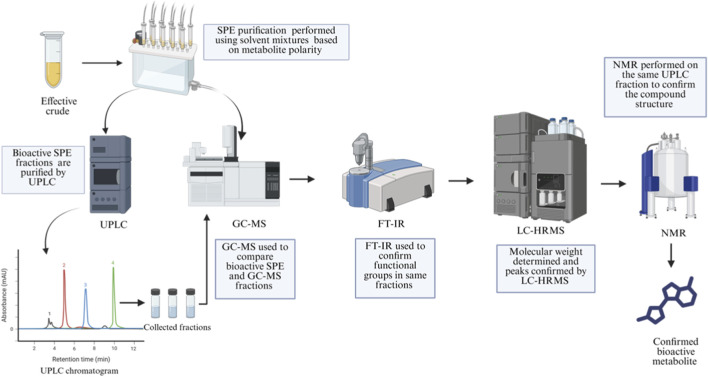
Schematic diagram representing downstream process workflow in endophytic research. Created in BioRender. Osborne, J. (2026) https://BioRender.com/x0xnmse.

Following fermentation, downstream processing is a critical phase of bioproduct development comprising extraction, purification and identification of bioactive lead molecules from microbial, plant and animal sources. In addition to lead molecule recovery, it also focuses on optimal resource use and reduced waste generation thereby abiding to sustainable bioprocessing practices (Yasu, 2024). In endophytic research, downstream processing involves sequential use of high throughput technologies as depicted in Figure 3 to isolate, purify and concentrate the lead molecules of interest, but often becomes challenging due to differences in polarity, solubility and stability of different metabolites. To maximize recovery of target molecules, extraction strategies must be carefully optimized to minimize losses and degradation. Successful protocols combine optimized sampling, rapid quenching and tailored extraction methods. For instance, quenching within ≤1s is generally recommended to preserve the properties of highly liable metabolites (Liu et al., 2019).

Distinct protocols are applied for intracellular and extracellular metabolite extraction. Intracellular extraction is a multistep procedure comprising sample harvesting, rapid quenching of metabolite activity and subsequent solvent extraction while a more direct metabolite recovery consisting of cell separation through filtration followed by extraction from culture medium (Mohd Kamal et al., 2022). As secondary metabolites are produced only in trace quantities and constitute only a smaller fraction of microbial metabolome, it is important to carry out structural elucidation of target metabolite prior to large scale downstream processing. This step enables the identification of potent metabolites, circumvent previously characterized compounds and prevents redundant rediscovery and avoids undesirable molecules, thereby streamlining the recovery process (Zhang R. et al., 2025).

Rediscovery of already known compounds is a major problem leading to the ruining of several factors which include resources and time. Dictionary of Natural Products (DNP) has recorded almost 340,000 natural products till the current edition, hence, effective dereplication strategies should be adopted to reduce rediscovery. Dereplication is a process of early detection and replacement of already discovered bioactive metabolites which can be achieved by integrating natural product databases with spectroscopic analysis techniques (Pérez-Victoria, 2024) like GC-MS, LC-HRMS, NMR, etc. These analytical techniques can be integrated, and the resulting spectral data can be compared to identify if the compounds of interest have already been discovered. Therefore, dereplication strategies involving integration of multiple analytical techniques are more efficient in identifying known compounds before investing time and resources into their purification. Bioinformatic tools like DAFdiscovery have been introduced to compare datasets obtained from NMR and MS using covariance and correlation analysis (Borges and Teixeira, 2024). Reports also indicate the increasing application of tandem spectroscopy (MS/MS) for generating fragmentation patterns of metabolites which are compared using molecular networking tools like CLMN (Classical Molecular Networking) and FBMN (Feature Based Molecular Networking). These methods compare similar MS/MS data and cluster them as networks which can be matched with spectral libraries to identify known compounds (Qin et al., 2022) thereby aiding in time management by eliminating the known compounds at the earliest.

### 4.1 Extraction of effective metabolites: focusing on the widely employed liquid-liquid extraction approach

Liquid-liquid extraction (LLE) is the most widely adopted sample preparation technique in endophytic research because of its simplicity, flexibility and efficacy in separating compounds. This sample preparation technique allows to preconcentrate and clean up the samples before further analysis and is based on the potential of solutes to distribute themselves between two immiscible liquid phases (Tay and See, 2025; Pramanik et al., 2024). Basically, LLE is carried out in three sequential steps from mixing of feed with the suitable solvent followed by phase separation to recovery of the solvent and discarding or recycling of the feed (Klemz et al., 2021).

Solvent recovery can effectively be done once solvent selection is appropriate. Typically, a range of solvents ranging from non-polar to polar (ethyl acetate, chloroform, methanol, dichloromethane and hexane), are used to target exact metabolite classes (Buragohain et al., 2023; Soliman et al., 2022; Santra et al., 2022). Messina et al. (2021) conducted an extensive study on *Posidonia oceanica* beach-cast leaves and pointed up the importance of choice of solvent and its influence on metabolite recovery. LLE proceeds with a workflow that typically initiates with centrifugation of mass multiplied or fermented broth to remove microbial biomass (5000rpm for 30 min) which is followed by mixing up of the clear supernatant with an equal volume of chosen organic solvent. The mixture is then incubated at low temperature (4 °C) to improve phase separation and reduce chances of degradation. Subsequent steps include solvent separation via a separating funnel and concentration of organic phase using a rotary evaporator. This is often followed by redissolution of dried crude in predetermined minimal volume of same solvent for further processing or in DMSO for storage (Deka et al., 2017; Nxumalo et al., 2020).

Several researchers have investigated modified LLE protocols to enhance metabolite yield and efficacy of extraction. Fanoro and Oluwafemi (2025), performed LLE with the help of ethyl acetate and chloroform in 1: 1 ratio, and the recovered extract was concentrated using a vacuum evaporator at 55 °C, while Kumari et al. (2021) employed a similar approach and performed triple extraction of fungal broth using ethyl acetate taken in equal volumes and the recovered extract were concentrated in a rotary evaporator at 65 rpm and 40 °C, with the final drying step in a fume hood to ensure gradual solvent evaporation and to ensure preservation of thermolabile compounds. A more sophisticated methodological comparison was reported by Ashitha et al. (2019), which included three distinct LLE protocols for bacterial endophytes. They compared the conventional method of mixing the culture broth and solvent followed by phase separation and centrifugation with thermal disruption through sequential heat shock (5 min in boiling water, followed by 5 min in cold water) and mechanical lysis of cells by sonication. Despite altering the entire set of sequence, a modification was applied only to pretreatment process leaving intact the downstream solvent separation and drying steps across all three methods the study demonstrated a tailored approach is required for different microbes to reap maximum benefits out of the extraction process.

Though LLE has been an efficient process for the extraction of lead compounds, it also has its own limitations. Conventional LLE is heavily biased towards the extraction of lipophilic or amphipathic molecules. This is due to the solvents that are employed in standard LLE and its importance in water immiscibility. Such solvents which are immiscible in water are generally non-polar to moderately polar in nature, thereby help in extracting hydrophobic compounds. Although co-solvents can be added to slightly modify the polarity (Drouin et al., 2018) the movement of highly polar metabolites from the aqueous to organic phase has been a major drawback. Therefore, LLE might lead to polarity dependent extraction bias making the hydrophilic bioactive molecules remain undiscovered. These limitations are overcome by modified LLE based methods like Dispersive Liquid-Liquid Microextraction (DLLME) using ionic fluids which are currently gaining importance. Xu et al. (2013) has reported the extraction of highly polar aminoglycoside antibiotics like gentamycin, tobramycin and neomycin using ionic fluid 1-hexyl-3- methylimidazolium hexafluorophosphate.

### 4.2 Partial purification of recovered metabolites by solid phase extraction (SPE)

SPE is recognized as an efficient method for separation, purification and concentration of analytes from various matrixes including crude extracts obtained from fermented broth (Buragohain et al., 2023; Badawy et al., 2022) The method employs a solid sorbent material which can be a cartridge, 96-well plate or a disc on to which the analytes adhere during the process. A careful selection of sorbent material is essential to elute out the target metabolite, and it is determined by the physicochemical properties of the target metabolite (Młynarczyk et al., 2024). Once the crude extract is passed through the sorbent, analytes become adsorbed or absorbed onto it and can be eluted subsequently using appropriate solvents. The resulting elutes termed as fractions are subjected to high throughput analytical techniques such as GC-MS, UPLC, LC-MS, FT-IR and NMR for further processing and identification (Manam et al., 2025b; Młynarczyk et al., 2024).

SPE when compared with classical separation and purification techniques like LLE, is more efficient in extraction and purification of antibiotics and bioactive lead molecules from diverse matrices because of its reduced time and solvent requirement, however, extraction of multiple analytes of both polar and non-polar nature remains challenging. These limitations are subdued by latest innovations in sorbent technology, expanding the grounds of SPE to another levels. Dispersive solid phase extraction (dSPE) and magnetic solid phase extraction (MSPE) are the two variants developed, along with modified sorbents like carbon nanotubes and molecularly imprinted polymers (MIPs) to improve extraction efficacy by focusing on targeted approaches (Suseela et al., 2023).

The separation mechanism in SPE is based on interactions between sorbent and analytes in solvent and is governed by factors like polarity, ionic charge, size and reactivity profiles of the analytes (Dugheri et al., 2021). SPE can be carried out in different modes, among which reverse phase chromatography-based SPE is most employed in endophytic metabolomic research proving its efficiency in processing crude extracts (Buragohain et al., 2023; Dey et al., 2023). Oasis Hydrophilic-Lipophilic Balance (HLB) cartridges are mostly employed for RPC-SPE in which copolymer of N-vinylpyrrolidone and divinylbenzene is used as the packing material for the retention of polar, non-polar and neutral compounds (Jeong et al., 2017) and the columns are subjected to a preconditioning with a selected solvent and are eluted under vacuum using gradients of solvents to maintain a range of polarities thereby achieving efficient metabolite recovery and separation (Buragohain et al., 2023).

### 4.3 Complete purification of the SPE fractionated metabolites by ultra-pressure liquid chromatography (UPLC) and high-pressure liquid chromatography (HPLC)

UPLC provides better performance in terms of resolution, speed and sensitivity when compared with HPLC (Taleuzzaman et al., 2015) The major demarcation between the two techniques lies in the particle size of the stationary phase, where the analytes adhere closely before separation. HPLC works with a particle size of 2.5–10 microns while UPLC allows a particle size often less than 2 microns (Nahar et al., 2020), thereby enabling shorter columns thereby minimizing solvent consumption. Furthermore, the smaller particle size improves the process of purification and aids peak quality but requires an appreciably higher pressure to operate exceeding 6,000 psi which is way too far from the highest limit of HPLC (5,800 psi). UPLC instruments are therefore tailored to withstand appreciably high pressures (14,500 psi) (Gupta et al., 2022). Underpinned by the high-pressure conditions in UPLC, it becomes difficult to maintain the retention time. This challenge is easily surpassed using polymeric columns and silica-based particles which withstand great pressures (Sheliya and Shah, 2013).

Investigations focused particularly on endophytic metabolites as discussed above used reverse phase columns (Acquity UPLC BEH C18) which is paired with a photodiode array detector (PDA) (Buragohain et al., 2023; Manam et al., 2025b). Almost across all the systems a stable flow rate of 1 mL/min is maintained. Moreover, chromatographic peaks are obtained, and individual fraction collectors can be used to collect respective peaks according to the retention time. Collected peak fractions can be used to confirm the antagonistic activity by drop plate method and thus fractions without any zones can be discarded thereby narrowing down the fractions with target metabolites for further analysis (Manam et al., 2025b).

### 4.4 Identification of target metabolites by GC–MS and LC–HRMS

GC-MS is one of the high throughput analytical techniques used to identify and analyze the compounds (metabolites) present in crude as well as fractions in endophytic research. It is best suited for volatile compounds separated by gas chromatography and can further be identified by mass spectrometry (Sriwastava and Srivastava, 2021; Samirana et al., 2022). Identification of compounds are achieved by comparing the similarity index (SI) and reverse similarity index (RSI) from the spectral data with already established databases like National Institute of Standards and Technology (NIST) (Santra et al., 2022).

GC-MS as discussed above focuses on volatile organic compounds (VOCs) and gaseous microbial metabolites (Isobe et al., 2011) behalf of which Bhuiyan et al. (2025) successfully isolated and extracted secondary metabolites from crude extracts of endophytic bacterium isolated from *Enhydra fluctuans*, while a similar approach was done by Bansal et al. (2025) reported the ability of GC - MS in detecting molecules even in nanogram quantities thereby highlighting the sensitivity the instrument holds. Also, it was noted that compounds present in trace amount often escaped detection thus marking a boundary for sensitivity of certain classes of metabolites like taxol.

On the contrary, LC - HRMS focuses on non-volatile organic microbial metabolites present within the crude extracts and are analyzed by comparing m/z values, retention time and expected molecular formula with established databases (Veilumuthu et al., 2024). Veilumuthu et al. (2024), reported the presence of Kendomycin from an endophytic strain (VITGV156) with a molecular mass of 470.3007 Da and a retention time of 3.63 min thereby portraying the importance of LC-HRMS as an analytical technique in identification and confirmation of a microbial metabolite having potent antimicrobial properties. A similar study was undertaken on fungal endophytes isolated from *Solanum mauritianum* and the effective bioactive metabolites were identified and confirmed using LC - QTOF - MS/MS and GC - HRTOF - MS. Ethyl acetate and methanol were selected for processing polar and non-polar solvents accordingly with the help of a reverse phase C-18 LC column with mobile phase supplied in gradient (water: acetonitrile). Workflow was completed by comparing databases such as PubChem, KEGG and MetFrag and reported the presence of various compounds (Ogofure and Green, 2025) thereby substantiating the importance of LC-HRMS in identification of microbial metabolite in endophytic research.

### 4.5 Confirmation of metabolites by FTIR and NMR and elucidation of functional groups and structural data

FT-IR is an extensively employed spectroscopic technique in endophytic research employed to confirm the expected metabolite based on fragmentation patterns thus revealing functional groups (Buragohain et al., 2023). FT-IR spectral data is of prime importance and is formed by the absorption of infrared radiation by the molecules at specific frequencies that generate bond vibrations (Kassem et al., 2023). For instance, Nischitha and Shivanna, (2022), confirmed the presence of antioxidant metabolites like ergosterol peroxide, solanine, cannabidiol, etc., by matching the FT-IR spectral data with the functional groups of expected compounds. Such comparative analysis between GC-MS identified compounds with FT-IR spectra can be used in confirmation of bioactive metabolites and results can be further substantiated by superimposing with respective UPLC chromatograms (Manam et al., 2025a). A related approach was reported by Yehia et al. (2020), where FT-IR spectra generated revealed the presence of functional groups such as C-H (697.90 and 931.94 cm^-1^), C=C (1,218.17 cm^-1^), C-O (1,301.41 cm^-1^), C=O (1,699.89 cm^-1^), CH_3_-C-H (2,928.44 cm^-1^) and OH (3,300 cm^-1^), which when compared with the GC-MS results revealed the effective compound present to be 3-phenylpropionic acid. Table 5 summarizes similar methods adopted in endophytic research in confirmation of effective metabolites.

**TABLE 5:** Recent studies focusing on FT-IR spectral data for confirmation of effective endophytic secondary metabolites.

Source	Endophyte	FT-IR absorption peaks (cm^-1^)	Functional groups	Probable metabolites	Remarks on activity	References
*Ziziphus mauritiana*	*Bacillus subtilis*	3,393.58	N-H	Gougerotin	Antibacterial; non- toxic (seed germination assay)	Manam et al. (2025b)
		1,636.70	C=O			
		1,440.22	Methylene			
		1,374.74	Methyl			
		1,037.83	Alkyl aryl ether			
		918.87	Alkene			
*Citrus limon*	*Bacillus sp.*	3,389.43	N-H	Pyrrolo [1,2 α] pyrazine-1,4-dione, hexahydro-3-(2-methylpropyl) (PPDHMP)	Antibacterial; First report from this host	Buragohain et al. (2023)
		2,257.96	C≡C			
		1,642.81	C=O			
		1,378.47	CH_3_			
		1,035.07	C-H			
*Myrancrodruon urundeuva*	*Pseudofusicoccum stromaticum*	3,320	N-H	cyclo-(L-Phe-D-Leu-L-Leu-L-Leu-L-Ile)	No cytotoxicity against HCT-116 (IC_50_ > 50 μg mL^-1^)	Sobreira et al. (2018)
		1712	C=O			
		1,648	Aromatic group			
		1,537	Aromatic group			
*Pteris pellucida*	*Emericella qaudrilineata*	1728	Carbonyl	Benzyl benzoate	Potential for combinatorial chemistry	Goutam et al. (2016)
*Gracilaria edulis*	*Bacillus subtilis*	3,380.99–1,034.726	N-H; C≡C; C=C; CH_3_; C-H	PPDHMP	Antibacterial; antibiofilm; beta- lactamase inhibitor; non-toxic	Manam et al. (2025a)
*Averrhoa carambola*	*Psychrobacter faecalis*	3,389.43	N-H	Noformicin	Antibacterial; non-toxic (seed germination assay)	Dey et al. (2023)
		2,257.96	S-H			
		1,642.81	C=C			
		1,378.47	O-H			
		1,035.07	S = 0			

Further confirmation of the identified metabolite is carried out by elucidating its structural characteristics for which NMR, a yet another powerful spectroscopic technique is employed (Shah et al., 2024). NMR, with its ability to work with electromagnetic radiation under strong magnetic fields, is considered a convenient technique with minimal sample preparation and consistent results with the ability to analyze metabolites even without standards. It elucidates the structure of metabolite by analyzing atomic nuclei of the metabolite and its interaction with high frequency radio waves and uses the signals from ^1^H,^13^C and ^31^P spectrum to produce the results (Zakariyah et al., 2024). There are softwares like DFT, which when combined with NMR provides accurate NMR calculation (^13^C) (DiBello et al., 2023). Additionally, hybrid approaches like SUMMIT MS/NMR combining NMR and MS data are being studied and used recently to accurately predict the structures. Also, the metabolite identification has advanced recently combining several high throughput techniques for and precise reproducible results. 3D NMR coupled with FT-ICR MS is recently gaining importance in metabolomic research (Boiteau et al., 2018).

Although significant advances have been made, studies demonstrated by Numan et al. (2022), effectively elucidated their metabolite structure using 1D and 2D NMR. Aligning with this Liu et al. (2023) used 1H and 13C NMR to effectively elucidate their metabolite structure. Considering these observations, it is high time for researchers to shift to advanced hybrid technologies to save time and get accurate results. Table 6 Provides information on all the high throughput techniques and its advantages and limitations.

**TABLE 6:** Overview of applications, advantages and limitations of major high throughput technologies.

Technique	Major application	Advantages	Limitations	References
HPLC	Identification, separation, purification and quantification of drugs, bioactive compounds	Highly sensitive; autosampler reduces manual workload; rapid separation (<30 min); reproducible; samples are recoverable	High-equipment cost; extensive sample preparation; high solvent consumption; troubleshooting requires skilled personnel; peak overlap; frequent column replacement	Shah et al. (2025); Shaikh and Uzgare (2026); Alqahtani et al. (2025)
UPLC	Separation and quantification of bioactive metabolites; detection of impurities; ADMET assessment	Higher sensitivity than HPLC; reduced analysis time (<90%) (short columns); enables quick switching of mobile phase and columns	Expensive instrument; frequent column replacement; smaller particle requires high pressure	Gumułka et al. (2022); Kiran and Madankar (2025); Yenugukesava and Dharmamoorthy (2025)
GC-MS	Separation and identification of compounds of environmental and pharmaceutical samples	Detection of volatile compounds; spectral databases aid compound identification	Unsuitable for thermolabile and non-volatile compounds; extensive sample preparation	Khalifea and Ali (2025); Ren et al. (2018)
LC-HRMS	Identification of compounds; confirmation of compounds using reference standards	Minimal sample processing	Decreased detection of trace compounds	Ren et al. (2018); Fabresse (2025); Lucci et al. (2017)
FTIR	Functional group identification of pharmaceutical samples; nanoparticle characterization	High accuracy; less expensive; non-destructive	Overlapping signals; highly sensitive to sample preparation; difficulty in analyzing non-polar compounds	Fahelelbom et al. (2022); Pasieczna-Patkowska et al. (2025)
NMR	Elucidates structure of bioactive compounds	Reproducible; non-selective; simple sample preparation; non-destructive; quantitative; rapid analysis (1D ^1^H-NMR)	Peak overlaps; poor sensitivity	Emwas et al. (2019)

## 5 Confirming non-toxic nature of endophytic metabolites via toxicity assays

Following lead molecule or bioactive metabolite identification, rigorous toxicity assessment is essential to rule out any chances of harmfulness to living cells and tissues (Islam et al., 2025). A metabolite as drug may become unsafe and cytotoxic due to several mechanisms like metabolic arrest, interference with cell proliferation or cell death. Therefore, effective toxicity assessment underlines the need for more assays rather than concluding from limited tests (Sazonova et al., 2022), pin pointing to the importance of carrying out *in vitro* as well as *in vivo* assays to ensure consistent, precise and unprejudiced results.

### 5.1 MTT and XTT assays for assessing endophytic metabolite toxicity

Tetrazolium assays are widely employed for confirming the non-toxicity of metabolites by quantitatively evaluating cell viability. These are preferred greatly by researchers because of the easiness to perform and interpret along with the reproducibility it provides (Madorran et al., 2024). The assays rely on cells ability to reduce the tetrazolium dyes via their biochemical activities. MTT, a yellow-coloured compound gets converted into an insoluble purple coloured formazan crystal in viable cells due to the activity of mitochondrial reductases. This colour change serves as the foundation for analysis where the insoluble purple formazan can be quantified spectrophotometrically (570 nm) after dissolving the crystals in suitable solvents thereby linking directly to cell viability (Kumar et al., 2018).

Consistent with this, XTT follows a similar assessment pattern and avoids formation of insoluble crystals as MTT assays thereby becoming an alternative modified version with no additional solvents required. Yellow coloured XTT gets converted to water soluble version or formazan in orange colour due to the activity of mitochondrial enzymes present in the cells. Spectrophotometric analysis corresponds to cell viability and is evidently a faster and sensitive assay but falls slightly to the expensive side (Kamiloglu et al., 2020).

MTT assay being widely employed in endophytic research, XTT also finds its space in literatures and investigations because of its advantages over MTT. For instance, Venkatachalam et al. (2024) determined IC_50_ value of fungal endophyte extracts using MTT assay. Similarly, Agustina et al. (2024) assessed the cytotoxicity of endophytic fungi from *Phaleria macrocarpa* to check toxicity profiles of their bioactive metabolite. Recent works by Nassar et al. (2023), and Naveen et al. (2023) also employed these *in vitro* toxicity assays for cytotoxic screening of their endophytic metabolites and nanoparticles.

### 5.2 Seed germination assay for toxicity valuation of endophytic metabolites

Phytotoxic nature of endophytic metabolites are studied employing seed germination assays since years. Seeds are selected according to the nature of study and different treatments such as cells of the isolate, supernatant, extracted crude along with respective controls are supplemented to seeds and germination is monitored to analyze qualitative changes often by measuring radicle and plumule lengths at specific intervals.

Several studies on endophytes including Manam et al. (2025a), Buragohain et al. (2023), and Dey et al. (2023) employed seed germination assay to evaluate the toxicity of the pathogen inhibiting isolate. Apart from endophytic metabolomic studies, Chakraborty et al. (2025a) reported successful use of seed germination in endosymbiotic bacteria obtained from horse milk, further proving the assay’s ability to generate consistent and trustworthy results in terms of toxicity linked with seed during the process of seed germination. Adding on to this, seed germination is a well established and reliable *in vivo* toxicity assay technique used to test whether the lead molecule or organism is toxic to the seeds and affects germination or not (Masangwa et al., 2017).

An isolate or a metabolite is considered non-toxic if the treated seeds exhibit plumule and radicle lengths and germination rate and growth compared to untreated control groups. Moreover, seed germination assays are widely employed in multidisciplinary fields to focus on phytotoxic responses. Investigations by Ranucci et al. (2024), followed this method for evaluating phytotoxicity, whereas Wagh et al. (2024) proved this method to be effective in testing phytotoxicity of heavy metals on different seed varieties. Collectively seed germination marks itself as a screening method for toxicity assessment in cross disciplinary studies.

### 5.3 Cell line toxicity assays for valuation of endophytic metabolites

Development of novel drugs should include early screening for cell line toxicity as several reports have documented post-marketing drug withdrawals due to unexpected toxic effects. Nephrotoxicity (Rao et al., 2022) and hepatotoxicity (Guo and van den Beucken, 2024), are the important toxicity challenges that must be addressed to prevent later stage withdrawals. Even if a drug is highly effective it will be withdrawn from the market if it causes toxic effects in humans. Therefore, hepatotoxicity is a red flag for drugs as every drug reaches the liver for metabolism and (Kim et al., 2025) hence, evaluation of drug induced hepatotoxicity in cell lines like HepG2, C3A and HepaRG are widely employed. HepG2 cells express nuclear transcription factors (Nrf2) which contribute to drug metabolism. However, they exhibit relatively low concentration of CYP enzymes limiting their drug metabolizing ability. C3A, a subclone of HepG2 expression has reported to be increasing the CYP enzyme concentration making them more suitable for assessing hepatotoxicity than HepG2 (Kim et al., 2025). Li et al. (2023) tested the hepatotoxicity effects of amiodarone using HepG2 spheroids. Instead of conventional 2D model, they employed a 3D model to exactly mimic the liver tissue which was administered and at a concentration of 50 µM for exposure periods ranging from 0.5–72 h. Parameters like cell death, dose response, time dependent response and histological tissue analysis were evaluated. The results reported that shorter exposure time is directly proportional to minimal or no cytotoxic effects, whereas prolonged exposure led to increased cell death. Therefore, assessing hepatotoxicity is essential prior to drug development and clinical progression.

Similar to liver, kidneys are also heavily susceptible to drug-induced toxicity. However, preclinical determination of nephrotoxicity of drugs still remains challenging (Tiong et al., 2014). HEK293 has been widely used as a model for nephrotoxicity assessment of drugs. Coskun-Demirkalp et al. (2025) demonstrated the nephrotoxic nature of paracetamol using HEK293 cells. Study exposed HEK293 cells to varying concentration of drug for different time periods. Parameters like apoptosis, disturbances in cell cycle, colony forming ability and gene expression changes were recorded and identified that drug tested is predominantly associated with dose-dependent cytotoxic effects in HEK293 cells. Therefore, assessment of toxicity through cell line studies could pave way for the identification of a lead molecule being converted into a commercial drug.

### 5.4 Zebra fish as a model organism for rigorous toxicity assessment for endophytic bioactive metabolites

Acute toxicity of bioactive metabolites is extensively assessed using zebra fish embryos (Shen et al., 2023), because of their high similarity with human genome. It is reported that zebra fish genomes resemble almost 70% with human genome and about 80% of genes identified are associated with human diseases (Shen et al., 2023; Liu C. et al., 2024). Additionally, zebra fishes hold immune systems with innate and acquired immune responses that relate closely with human systems, thereby holding a space in toxicological research and immune system disorders (Mandrekar and Thakur, 2009). Furthermore, the zebra fish embryos are optically transparent allowing instantaneous observation of toxicological effects or organ formations. Moreover, these embryos can be subjected to repeated trials effortlessly and do not require any ethical clearance as they are not grouped as animals until 5–6 days post fertilization as per EU Directive 2024/1262 (Aschner et al., 2025).

Zebra fish embryos are cultured using embryo medium in 96 well plates and treatments along with control are supplemented for predetermined periods. Usually, an incubation period of 24–48 h at 27 ± 2 °C reveals morphological and phenotypical changes and LC_50_ can be determined (Mandrekar and Thakur, 2009). Table 7 reviews selected studies on zebra fish related toxicity assessment particularly focusing on endophytic metabolites and purified compounds with an increased focus on endophytic fungus rather than endophytic bacteria. Most of the studies include negative controls while often fail to include positive controls for precise conclusions thus hindering direct comparisons across studies. In general, zebra fish models are sensitive and ethically favorable for preliminary toxicity analyses, and the toxicity assessment can be furthermore endured by including positive controls. Endophytes and endophytic bacterial metabolites are to be included to tighten the pillars of toxicity assessments.

**TABLE 7:** Overview of zebra fish toxicity assessment in endophytic research.

Bacteria	Source category	Concentration of extract/Bacterial suspension exposed to	Test substance	Exposure period	Zebra fish stage used	Control	Specific toxicological endpoints measured	References
*Phytobacter* sp	Endophytic bacteria (*Oryza sativa* seeds)	1 × 10^9^ CFU/mL, 1 × 10^10^ CFU/mL, 2 × 10^10^ CFU/mL (for non- lethal dose)	Bacterial suspension (grown in TSB, resuspended in PBS)	7 days (5 days exposure, 2 days without bacterial exposure)	3–6 months aged zebra fish - adult	Untreated system water (negative control), fishes in 1X PBS (vehicle control)	Survival assessment by kaplan-meier survival assay	Jana et al. (2025)
*Phytobacter sp*	Endophytic bacteria (Oryza sativa seeds)	50 μg/mL, 100 μg/mL, 250 μg/mL	Pure indole dimer antifungal metabolite	3 days	Embryos (24–96 hpf)	0.1% DMSO in E3 medium	Survival assessment by kaplan-meier survival assay; developmental toxicity; biocompatibility assessment; intestinal localization by fluorescence imaging	Jana et al. (2026)
*Citrobacter freundii; Bacillus subtilis; Pseudomonas otitidis; Burkholderia cenocepacia*	Endophytic bacteria (Curcuma zedoaria)	0 ppm, 50 ppm, 100 ppm, 150 ppm, 200 ppm, 250 ppm	Crude in ethyl acetate and aqueous phase extract with bacterial metabolites	72 h	Fertilized zebrafish embryos	0 ppm extract treated embryos	LC50 determination, embryo mortality, coagulation, hatching failure, developmental abnormalities, body axis changes, somite abnormalities, pigmentation changes, yolk sac defects, eye and cardiac defects and impaired blood circulation	Sulistiyani et al. (2019)
*Fischerella sp*	Non-endophytic bacteria	12-Epi-hapalindole H isonitrile): ≥5 μg/mL; 12- Epi-ambiguine B nitrile: ≥10 μg/mL; Crude fractions: ≥10–250 μg/mL (depending on fraction)	Purified compounds; extracts; fractions from chromatography	5 days post fertilization (dpf)	4–32 cell stage embryos (<6hpf)	Not clearly specified in the study (vehicle control used same solvent without test compound)	Teratogenicity (pigmentation loss, body axis curvature, pericardial edema); recovery after removal, dose dependent developmental toxicity	Walton et al. (2014)
*Phormidium nigroviride*	Non-endophytic bacteria	100 μg/mL, 120 μg/mL, 160 μg/mL, 200 μg/mL, 250 μg/mL	Bacterial crude extract dissolved in DMSO	0–120 hpf	Embryos ≤5 dpf	Blank control (reconstituted fish medium); solvent control (DMSO (≤0.1%))	Developmental defects (spinal deformities, pericardial edema, yolk sac edema, eye size reduction); reversibility of morphological abnormalities; embryo viability	Davidović et al. (2025)

## 6 Efficiency assessment of API by analyzing pharmacokinetic and pharmacodynamic parameters

Development of a bioactive metabolite which can be termed as an active pharmaceutical ingredient (API), becomes pointedly more efficient when the PK and PD properties of metabolites discovered are well understood (Palmer et al., 2022). Initially, PK was used to recognize the path through which a drug moves in, remains and moves out of the body while PD was used to understand the reaction mechanism of body against a particular drug concentration. Over the years, pharmaceutical research has advanced and attempted to combine both the PK and PD concept and introduced PK/PD modelling which helps to elucidate dose-response relationships via framing mathematical models, thus aiding to increased efficacy of the drug designed. Such an approach paves way for personalized treatments thus coming up with realistic and reasonable doses thus improving clinical reasoning (Pereira et al., 2022).

PK/PD analysis has become mandatory for a novel metabolite to be considered as a drug (Ranjani and Hemalatha, 2021). The analysis sheds light on absorption characteristics of metabolite to cross various barriers like skin, blood-brain barrier (BBB), and gastrointestinal (GI) tract and ensures efficacy and safety by ruling out the possibility of undesired metabolic interactions. With the advancement of technology, softwares like SwissADME, Molsoft, ADMETlab 2.0 etc., have been developed to predict the ADME (Absorption, distribution, metabolism, and excretion) properties of novel metabolites (Tharani and Rao, 2024).

Following a successful elucidation of an effective metabolite, efficiency evaluation should start from *in silico* approaches and then move gradually to animal models. *In silico* approaches begin with analyzing the ligands and elucidating the compound structures. For instance, Ranjani and Hemalatha, (2021), employed SwissADME to analyze the endophytic fungal ligands extracted while Bhowmick et al. (2025) employed SwissADME along with pkCSM to analyze metabolites extracted from *Aspergillus fumigatus*. Further the compound structures can be obtained with the help of softwares like ChemDraw Professional 16.0 and can further be analyzed by changing to SMILES. Studies revealed that such workflows aid in prediction of parameters of importance like water solubility, CYP450 inhibition, hepatotoxicity and Lipinski’s Rule of Five making PK/PD analysis as an inevitable process in API development.

While *in silico* ADMET tools rapidly filter candidate molecules, many highly successful natural product antibiotics (Vancomycin, Erythromycin) violate Lipinski’s Rule of Five due to their large molecular weights and complex macrocyclic structures. Similarly, Cyclosporin A is a well-known orally administered drug that violates the rule of five but still exhibits bioactivity inside the human system. This is attributed to its ‘chemical chameleon’ nature which helps it dissolve in aqueous fluids and reduce polarity while crossing lipid membranes (Lipinski, 2016). Therefore, over reliance on strict algorithmic filtering may prematurely discard potent, atypical natural APIs.

PK parameters such as drug clearance, volume of distribution, half-life of the drug (t^1/2^), maximum serum concentration (C_max_), time to C_max_ (tC_max_), area under serum concentration (AUC) have to be compared along with PD parameters like MIC of the target pathogen, E_max_ (maximum effect), and EC50 (concentration to achieve 50% of E_max_) to integrate PK/PD data for effective conclusions (Alikhani et al., 2025; Ding et al., 2026; Asín-Prieto et al., 2015).

## 7 Role played by pathogenic islands (PAI) and importance of targeting bacterial virulence

Pathogenic bacteria foster an array of mechanisms to acclimatize and thrive in adverse host environments. This might be by causing simple to harmful infections, or by supporting disease progression, all acquired as a result of genomic plasticity of the bacterial genome via horizontal gene transfer. For instance, pathogenic islands, symbiosis islands, and antibiotic resistance islands are all a part of bacterial genomic islands which pave way for bacterial evolution leading to extreme adaptation (Soares et al., 2012; Munshi et al., 2025).

Focusing more on pathogenicity islands underlines its importance in bacterial virulence. The presence of exceptionally large genomic islands harboring virulence genes obtained from other organisms helps the respective pathogen with properties like adhesion, colonization, invasion and immune system circumvention as well as production of toxins (Soares et al., 2012). This directly demonstrates the role of PAI in bacterial virulence and investigations in this field successfully prove that a pathogen loses its pathogenic potential when the virulence genes encoded PAIs are deleted from the genome. For instance, Wiktorczyk-Kapischke et al. (2023) through their studies demonstrated that deletion or inactivation of LIPI-1 pathogenicity island from *Listeria monocytogenes* resulted in overall loss of virulence. The work further substantiates the importance of focusing on more than one conserved island as leaving accessory PAIs may reflect in potential danger if proper investigations are not carried out for the entire PAI network.

Direct reports on endophytic metabolites neutralizing PAIs are limited, several endophytic metabolites have been shown to inhibit quorum sensing (QS) pathways. QS systems can regulate the expression of virulence associated and pathogenicity associated genes (Pena et al., 2019). Therefore, quorum sensing inhibition may indirectly suppress pathogenicity associated virulence expression leading to reduction of pathogenicity. Zeng et al. (2022) reported that Actinomycin D extracted from an endophytic bacterium (*Streptomyces cyanochromogenes* RC1) inhibited quorum sensing activity in *Pseudomonas aeruginosa* which resulted in less virulence factor production and biofilm formation thereby reducing bacterial pathogenicity indirectly. Also, bacterial endophytes are reported to have different secretion system (Chakraborty et al., 2025a). Aurodox is a natural bacterial metabolite that is reported to suppress type III secretion system in bacteria by particularly inhibiting the ler gene of LEE PAI providing strong evidence to effective anti-virulence strategies of bacterial metabolites by targeting the PAI’s which can be further explored by investigating more on endophytes (McHugh et al., 2019).

Advances in pharmaceutical research focuses increasingly on targeting bacterial virulence and virulence factors typically encoded within the PAIs rather than just focusing on inhibiting pathogenic growth and proliferation. New era is shifting towards anti-virulent agents showing broad spectrum activity by neutralizing the effects of virulent genes (Barczak and Hung, 2009). Endophytic research is progressing at a faster rate in a quest to discover novel metabolites with broad spectrum anti-virulent properties.

## 8 Importance of integrating artificial intelligence (AI) and machine learning (ML) in therapeutic research

Classical approaches on drug development have always been a labor-intensive and time-consuming process requiring high-level expertise before the onset of AI and ML models. Integrative approaches combining these models is the pressing need of the hour because of increased economic burden coupled with high failure rates and time constraints (Visan and Negut, 2024; Zhang K. et al., 2025). Ability of AI to mimic human brain in cognitive functions is exploited in therapeutic research to solve real time problems within a short period of time without multiple trials. These include drug target site identification, screening various compounds, optimizing media without many repeats and trials, thus reducing the number of experiments thereby saving time. Compared to conventional methods, AI tools have demonstrated to show increased efficiency with reported timeline reductions in drug developmental pipelines by approximately 1–8 years (Dhudum et al., 2024). High-throughput technologies like GC-MS, FT-IR, NMR and LC-HRMS confirm the lead molecule but often is associated with unclear hits and less expanded library. AI tools like GNPS (Global Natural Products Social Molecular Networking) helps to search compounds within a regularly updated vast datasets which helps to identify known compounds within no time. However, these can’t replace conventional high-throughput technologies, but it fastens the research work by help predict the possible structures in case of unknown or novel compounds (Muthuraj and Chandrasekaran, 2026). In contrast, AI tools like CASE (Computer–Assisted Structure Elucidation), can elucidate the structures of metabolites mimicking NMR data. Similarly, tools like SMART 2.0 and SMART-Miner and COLMAR have reportedly contributed to identifying novel compounds from NMR data (Mullowney et al., 2023). Identification of hidden BGCs within microbial genomes is often challenging, but with the integration of AI based ML models such as DeepBGC and PRISM4 has significantly contributed to efficiency. These are reported to predict structures of metabolites and their biological actions which can aid in preliminary screening of isolates with targeted activity thereby reducing time and effort associated with screening unpromising isolates. However, considering the predictive nature of these *in silico* approaches, confirmation should rely solely on experimental validation. *In silico* tools like DeepBGC, ClusterFinder, TOUCAN, BGC-Prophet, and PRSIM4 have significantly accelerated the prediction of BGCs and putative metabolite structures in natural compounds, in spite of several limitations. Many predicted BGCs remain silent or poorly expressed under laboratory conditions (Chigozie et al., 2025; Rutledge and Challis, 2015; Kawano et al., 2026). Therefore, the endophyte studied might also fail to produce predicted metabolite, requiring advanced techniques for expressing the bioactive genes such as cloning, BGC refactoring and heterologous expression (Xu et al., 2022). Furthermore, computational models can only provide predictive insights and cannot confirm metabolite production, biological activity, toxicity and proper structure stating the importance of experimental validation including structural elucidation, expression analysis, bioactivity screening and toxicity assessments. Thus underlines the fact that computational tools cannot replace experimental investigations, but can significantly reduce the time, expense and effort involved in screening metabolites that may ultimately lack biological activity.

Integration of AI and ML models in endophytic therapeutic research has significantly enhanced the efficiency of upstream processing, especially in effective strain identification and downstream processing like optimization of media and metabolite identification and characterization. Metabolite identification and characterization is often challenging due to spectral libraries and datasets; however, predictive models help reduce this workload. In their absence, conventional identification and characterization of metabolites require repeated trials, often with low metabolite yield and limited crude which further leads to repeated experimentations. Furthermore, the study of novel metabolites can be accelerated by excluding already reported compounds within a given isolate. Following *in silico* analysis, subsequent experimental validation can reduce number of trials, thereby facilitating the identification of a greater number of endophytic metabolites with broad-spectrum activity while minimizing effort and rial and error approaches.

AI-ML model efficacy in metabolomic research can be enhanced by the scientific community depositing their generated datasets (LC-MS/MS and NMR) to open access repositories such as MetaboLights and GNPS (Global Natural Product Social Molecular Networking). The data deposited can be raw data, metadata, metabolite structures, methodological details and bioactivity information across diverse species. This will help in advancing omics-based research where deposited high quality experimental data can be used to train ML models and compare with existing datasets to draw effective conclusions (Yurekten et al., 2024).

MetaboLights basically is a metabolomic data repository that enables deposition, storage and recovery of metabolite related datasets which has witnessed a significant growth in first half of 2025 with 438 complete studies and 1,467 ongoing studies. This system provides users with a permanent identifier only after validation to ensure data quality before acceptance (Thakur et al., 2026). The stored data can be downloaded, reanalyzed, and compared across multiple datasets using external analytical tools. On the other hand, GNPS is a molecular networking platform which allows both deposition and online analysis of metabolite datasets (MS/MS based). It also helps to match similar metabolites by comparing similar spectra for the identification of relevant related compounds within the deposited datasets. It promotes collaborations across the globe and helps in the identification of novel metabolites and novel chemical entities thereby supporting dereplication (Gaudencio et al., 2023; Salek et al., 2013). GNPS2 is an advanced version of GNPS which offers a high processing speed, user friendly interface and better analysis tool supporting bioactive metabolite identification from *in vitro* experiments. It also facilitates reverse metabolomics, where metabolites identified from lab experiments are searched against human metabolomic datasets to determine if similar metabolites are identified *in vivo* in humans. Thus, this enables an assessment on translational relevance of *in vitro* findings and helps predict whether metabolite formation observed in experimental systems are applicable to humans (Yu et al., 2026).

## 9 Discussion and implications

Medicinal plants and seaweeds are well recognized for their ability to produce metabolites with potential for translation into APIs. Traditionally, these sources have been exploited in natural product research; however, endophytes are increasingly gaining attention due to their advantages over direct extraction of lead molecules from host plants. Accuracy in upstream and downstream processes is crucial for maximizing results and minimizing errors. The discussion highlights the key limitations with errors and future prospects thereby highlighting the translational bottleneck persisting in endophytic therapeutic research.

### 9.1 Translational bottleneck and ‘valley of death’ in endophyte - based drug discovery

Endophyte-based drug discovery while progressing towards advanced stages experience several translational bottlenecks and several promising bioactive metabolites succumb to ‘Valley of Death’. Most of the investigations prioritize antibacterial properties of metabolites like MIC and assume that strong *in vitro* ability is directly related to its pharmaceutical potential. Such metabolites performing well under *in vitro* conditions often are reported to fail in *in vivo* studies. Several metabolites showing *in vitro* activity may exhibit poor bioavailability, rapid serum protein binding, or extreme host toxicity. Such failures occur when pharmacokinetics and pharmacodynamic properties are considered with less importance during early stage of screening. The mechanism by which the metabolite acts against the pathogens is important along with its ability to enter the body, be absorbed, reach the target site, and exert its effect within the human body is equally crucial (Sadgrove and Jones, 2019). Integrating pharmacokinetic, toxicity and chemical property assessment during the early stages along with antimicrobial screening might help reduce the final stage failure rates and improve translational efficiency.

Another fundamental limitation of current upstream processing is the removal of the endophyte from its host. Understanding the ecological trigger such as the reason behind host-microbe interaction for the defensive symbiosis often reveals the specific environmental stressors required to unlock optimal secondary metabolite production *in vitro*. These factors may be internal or external, hosts’ geographical location, season of sample collection and climatic conditions. Collectively these influences pinpoint that endophytes do not consistently produce identical bioactive compounds under laboratory conditions unless appropriate ecological stress signals are not replicated (Li et al., 2022).

API research often faces rapid rediscovery challenges which slow down the drug discovery pipeline by wasting significant time and resources on previously characterized compounds instead of focusing on novel bioactive metabolites. Dereplication can be employed as an effective strategy for the early identification of known metabolites, thereby redirecting research towards unidentified compounds. Integration of multidisciplinary dereplication strategies like Q-TOF-MS/MS, LC-MS/MS and ESI-TOF-MS, and high-resolution NMR technologies often help in enhancing metabolite stability and structural characterization early in downstream processing. Recent advances including imaging mass spectrometry (IMS), omics-based technologies, and public databases such as MarinLit and NMRShiftDB have accelerated the dereplication process by enabling rapid identification of known metabolites and their properties at early stages (Gaudêncio and Pereira, 2015).

Another recurring challenge is the inconsistency in metabolite production and recovery when endophytes are transitioned from their native host and associated environment to laboratory-based environment and bioreactors. This variability is likely attributable to the absence of host-mediated regulatory cues and disturbances in microbial communication networks that control secondary metabolite biosynthesis. Although techniques to mimic host and environmental dynamics have been proposed to resolve these problems, still reproducibility and scalability of the metabolite remain a persistent problem (Madagundi, 2025). Also, fermentation of microbes will produce very little metabolites in lab scale conditions with which researchers find it difficult to carry out all the high throughput studies. Here, shelf life and storability of the effective metabolite in the crude also poses a significant challenge. Optimization of culture media for precision fermentation is an effective approach for enhancing the production of specific metabolites and improving yield. However, manual optimization is often labor intensive due to highly interconnected metabolic pathways (Aida et al., 2023). Incorporation of ML can simplify this process by utilizing large datasets generated through high-throughput technologies to predict optimal macro-and micronutrient combinations for maximum metabolite production.

Additionally, prolonged and continuous subculturing of effective endophytic strains has been reported to reduce their metabolite producing ability completely or partially. This decline in bioactivity is often attributed to the absence of natural host-associated environmental conditions during axenic conditions (Deepika et al., 2020). Here, AI and ML assisted approaches coupled with metabolomics and transcriptomics strategies and optimization of culture conditions might help predicting the elicitors required in restoring or enhancing metabolite production.

Further challenges arise during scale up from flask level production to bioreactor systems, where metabolite yield and production rates are often hindered by limited understanding of biosynthetic pathways. This is further compounded by restricted availability of advanced gene editing tools tailored for endophytes which ensure scalability, and reproducibility to endophyte derived metabolites (Alum et al., 2026). Also, fermentation of microbes will produce very little metabolites in lab scale conditions with which researchers find it difficult to carry out all the high throughput studies. Here, shelf life and storability of the effective metabolite in the crude also poses a significant challenge.

Coupled with limitations, methodological errors further divert endophytic drug discovery from its core objectives. Surface sterilization is the primary and basic step which should be performed with utmost care and precision. Following surface sterilization protocols without keeping proper controls might lead to misidentification of epiphytes as endophytes. Also, while narrowing down the vast list of metabolites present in crude to the effective metabolites from GC-MS library, chances for identifying column compounds as effective metabolites are seen to be more. Furthermore, studies often limit bulk compound identification rather than narrowing down to effective metabolites and elucidating their structures so that precise data can be generated.

## 10 Drug discovery roadmap – scientific examples

Endophytic bacteria to pure compound synthesis were successfully demonstrated by Manam et al. (2025a) by investigating marine endophytic bacterium from *G. edulis*. The effective isolate was screened for antibacterial activity against selected human pathogens and was further biochemically and molecularly characterized. LLE was used to extract the bioactive compound and further purification of crude was carried out using SPE. Fraction with maximum bioactivity was analyzed using UPLC, GC-MS, and FT-IR. The lead molecule was identified structurally using NMR spectroscopy. PPDHMP was confirmed as the lead molecule, and molecular docking was carried out to predict the mechanism of action of the metabolite followed by toxicity assessment.

Buragohain et al. (2023) reported the presence of PPDHMP from endophyte of *Citrus limon* also illustrates on a single molecule for inhibiting pathogens. The lead molecules were identified through abovementioned high throughput technologies further validating the proposed roadmap. Tanvir et al. (2024), further demonstrated identification of BagremycinA by endophytic bacteria and reported to show antibacterial activity against human pathogens. Presence of BagremycinA was confirmed by comparison with HPLC-DAD-UV-VIS database.

## 11 Materials and methods

### 11.1 Literature search strategy and data procurement

PICO method was employed for framing research questions and for conducting a comprehensive literature search. Google Scholar, PubMed, and Web of Science were the electronic databases employed for extensive literature search in the relevant field. To precisely search topics and procure data, keywords related to the topics were used. Bacterial endophytes, upstream processes, downstream processes, multi-omics approaches, bioactive metabolites, toxicity assessment, pharmacokinetics and pharmacodynamics are few examples. Further clarity of search was enhanced by using Boolean operators (AND/OR) to combine keywords for retrieving related data. Articles published between 2023 were given primary importance followed by 2020–2022. However, articles published earlier were considered only when recent references failed to address the relevant topic.

### 11.2 Exclusion criteria

Irrelevant reports, conference proceedings and non-English publications were avoided. Also, studies lacking insufficient experimental data, those focusing only on phytochemicals with no note to endophytic bacterial metabolites were excluded. Retracted papers were avoided and papers that did not give clarity with the related topic were not considered (Chakraborty et al., 2025b).

## 12 Future prospects

An API derived from natural sources like endophytes serves as sustainable alternatives to antibiotics prevailing in market, however their therapeutic efficiency can be increased by achieving broad-spectrum activity. Gene editing tools engineered solely for endophytes are expected to open greater opportunities in endophyte-based drug research. CRISPR/Cas9 systems for targeted gene editing offers promising opportunities in endophytic bacterial research for enabling broad spectrum activities and increased metabolite production in effective isolates. However, the application of CRISPR/Cas9 systems on non-model organisms remains challenging due to several reasons. Lack of standardized protocols, limitations in homologous recombination and transformation, stability in Cas9 expressions, limited availability of suitable plasmids that may pause the developmental pipeline. Furthermore, works carried out in unculturable bacteria again complicate genetic modifications (Volke et al., 2023). Recent investigations in endophytic fungi include CRISPR/Cas9 systems for targeted gene editing (Verma et al., 2023), thus enabling broad spectrum activities and increased metabolite production in effective isolates. On contrary to this, much genetic engineering works are not carried out in endophytic bacteria leaving a void space in research. Also, investigations point out that studying the epigenetics of the effective isolate will help in modifying the biosynthetic gene clusters (BCG) thereby increasing the metabolite yields of the effective isolates. Integration of multi-omics technologies like genomics, transcriptomics, proteomics and metabolomics can effectively reveal the unrevealed molecular potentials of endophytes to produce bioactive secondary metabolites (Shishodia et al., 2025). Such approaches contribute to prediction and validation of compounds produced by bacteria through identification and analyzing the potential BGCs. Such approaches are crucial for exploring the metabolic potential of unculturable bacteria (Arini et al., 2025). Genomics enables prediction of potent BGCs within the genome of an organism. Upon activation of these BGCs, transcriptomics provides insights into expression of associated gene clusters. Proteomics further confirms the production of proteins or enzymes through biosynthetic pathways and metabolomics helps in the validation of the predicted bioactive metabolites synthesized by a biological system.

## 13 Conclusion

The increased failure of antimicrobials in pharmaceutical industries and healthcare sectors due to ever rising AMR and MDR patterns, have raised the demand for novel natural products as APIs to several folds. However, identification and designing of such a novel compound to a drug often becomes a cumbersome process requiring extensive toxicity assessment and clinical testing. A breakthrough in pharmaceutical industry thus requires effective upstream and downstream workflow to isolate, extract, purify, narrow down and identify the compound of interest. Coupled with this, there is a high need to address factors that prevail as challenges like reproducibility, instability and loss of bioactivity during the standardization process.

Endophytes have been a central focus for decades and have been reported to aid novel natural product discovery. On the contrary, literature suggests that more than half of novel metabolites identified so far used endophytic fungus thus leaving endophytic bacteria underexplored. Considering this as a potential void for novel discoveries endophytic bacterial metabolites hold potential for API development.

With the advances in natural product research, the microbial metabolites identified are subjected to PK/PD modelling to avoid method failure and capital loss during final stages, highlighting the requirement of multiple trials during the transition of an active drug from lab scale to clinical scales. Future API development must transcend isolated metabolomics. Integrating transcriptomics and proteomics (multi-omics) during the fermentation stage is essential to map the real-time regulatory networks governing BGC expression, allowing for the rational, targeted engineering of endophytes as hyper-producing microbial cell factories.

In synergy with focusing on bacterial inhibition by retarding proliferation, targeting PAI’s to aim virulent factors and encoded genes is the future. Combining endophytic research which offers biofilm inhibition and quorum quenching along with blocking toxin production and inactivation with deletion of target PAI’s holds promising future. Also, CRISPR technology even after considering its limitations, successful implementation could assist in combining the inhibitory effects of endophytes on different pathogens by gene editing to enable broad spectrum activity in a single isolate, thereby designing a magic bullet.
